# Hierarchical Modeling of Activation Mechanisms in the ABL and EGFR Kinase Domains: Thermodynamic and Mechanistic Catalysts of Kinase Activation by Cancer Mutations

**DOI:** 10.1371/journal.pcbi.1000487

**Published:** 2009-08-28

**Authors:** Anshuman Dixit, Gennady M. Verkhivker

**Affiliations:** 1 Graduate Program in Bioinformatics and Center for Bioinformatics, The University of Kansas, Lawrence, Kansas, United States of America; 2 Department of Pharmaceutical Chemistry, School of Pharmacy, The University of Kansas, Lawrence, Kansas, United States of America; 3 Department of Pharmacology, University of California at San Diego, La Jolla, California, United States of America; National Cancer Institute, United States of America and Tel Aviv University, Israel

## Abstract

Structural and functional studies of the ABL and EGFR kinase domains have recently suggested a common mechanism of activation by cancer-causing mutations. However, dynamics and mechanistic aspects of kinase activation by cancer mutations that stimulate conformational transitions and thermodynamic stabilization of the constitutively active kinase form remain elusive. We present a large-scale computational investigation of activation mechanisms in the ABL and EGFR kinase domains by a panel of clinically important cancer mutants ABL-T315I, ABL-L387M, EGFR-T790M, and EGFR-L858R. We have also simulated the activating effect of the gatekeeper mutation on conformational dynamics and allosteric interactions in functional states of the ABL-SH2-SH3 regulatory complexes. A comprehensive analysis was conducted using a hierarchy of computational approaches that included homology modeling, molecular dynamics simulations, protein stability analysis, targeted molecular dynamics, and molecular docking. Collectively, the results of this study have revealed thermodynamic and mechanistic catalysts of kinase activation by major cancer-causing mutations in the ABL and EGFR kinase domains. By using multiple crystallographic states of ABL and EGFR, computer simulations have allowed one to map dynamics of conformational fluctuations and transitions in the normal (wild-type) and oncogenic kinase forms. A proposed multi-stage mechanistic model of activation involves a series of cooperative transitions between different conformational states, including assembly of the hydrophobic spine, the formation of the Src-like intermediate structure, and a cooperative breakage and formation of characteristic salt bridges, which signify transition to the active kinase form. We suggest that molecular mechanisms of activation by cancer mutations could mimic the activation process of the normal kinase, yet exploiting conserved structural catalysts to accelerate a conformational transition and the enhanced stabilization of the active kinase form. The results of this study reconcile current experimental data with insights from theoretical approaches, pointing to general mechanistic aspects of activating transitions in protein kinases.

## Author Summary

Mutations in protein kinases are implicated in many cancers, and an important goal of cancer research is to elucidate molecular effects of mutated kinase genes that contribute to tumorigenesis. We present a comprehensive computational study of molecular mechanisms of kinase activation by cancer-causing mutations. Using a battery of computational approaches, we have systematically investigated the effects of clinically important cancer mutants on dynamics of the ABL and EGFR kinase domains and regulatory multi-protein complexes. The results of this study have illuminated common and specific features of the activation mechanism in the normal and oncogenic forms of ABL and EGFR. We have found that mutants with the higher oncogenic activity may cause a partial destabilization of the inactive structure, while simultaneously facilitating activating transitions and the enhanced stabilization of the active conformation. Our results provided useful insights into thermodynamic and mechanistic aspects of the activation mechanism and highlighted the role of structurally distinct conformational states in kinase regulation. Ultimately, molecular signatures of activation mechanisms in the normal and oncogenic states may aid in the correlation of mutational effects with clinical outcomes and facilitate the development of therapeutic strategies to combat kinase mutation-dependent tumorigenesis.

## Introduction

Protein kinase genes are signaling switches with a conserved catalytic domain that phosphorylate protein substrates and thereby play a critical role in cell signaling [1]–[5]. As a result, many protein kinases have emerged as important therapeutic targets for combating diseases caused by abnormalities in signal transduction pathways, especially various forms of cancer. A large number of protein kinase crystal structures in the free form and complexes with various inhibitors have been determined, resulting in the growing wealth of structural information about the kinase catalytic domain [6]–[9]. The crystal structures have revealed considerable structural differences between closely related active and highly specific inactive kinase forms [10]–[24]. Conformational plasticity and diversity of crystal structures of the ABL [10]–[21] and EGFR kinase domains [22]–[24] have demonstrated the existence of active, inactive, Src-like inactive and intermediate conformational forms. Conformational transitions and dynamic equilibrium between these distinct conformational states are important characteristics of the kinase regulation and recognition by other molecules [25]–[28]. Evolutionary analysis of the functional constraints acting on eukaryotic protein kinases (EPKs) demonstrated that protein kinase mechanisms may have evolved through elaboration of a simple structural component that included the HxD-motif adjoining the catalytic loop, the F-helix, an F-helix aspartate, and the catalytically critical Asp-Phe-Gly (DFG) motif from the activation loop. This computational analysis showed how distinctive structural elements of the kinase core may be linked with the conformational changes of the DFG motif in kinase regulation [29]. A surface comparison of crystal structures for serine–threonine and tyrosine kinases has recently identified the conserved residues that are most sensitive to activation [30]. According to the proposed model, critical features of the common activation mechanism may include a dynamic assembly of the “hydrophobic spine” motif and the formation of specific salt bridges that can collectively provide coordination of the kinase lobes during activation process [30],[31]. These illuminating studies have demonstrated that protein kinase function may be controlled by a dynamic assembly of spatially distributed conserved residues important in regulation of allosteric signaling pathways. In a subsequent study, it was proposed that the F-helix of the kinase domain may act as a central scaffold in the assembly of active protein kinase forms by anchoring the hydrophobic regulatory spine (R-spine) and a second functional cluster termed catalytic spine (C-spine) [32].

Abnormal activation of protein kinases is among major causes of human diseases, especially various cancers. Resequencing studies of kinase coding regions in tumors have revealed that a small number of kinase mutations contribute to tumor formation, while the majority are neutral mutational byproducts of somatic cell replication [33]–[35]. Mutations in protein kinases are implicated in many cancers [36] and often exemplify the phenomenon of oncogene addiction [37],[38], whereby structural effects of oncogenic mutations confer a selective advantage for tumor formation during somatic cell replication. The dominant oncogenes that confer the oncogene addiction effect include ABL, EGFR, VEGFR, BRAF, RET, and MET kinase genes [38]. The dependence of chronic myeloid leukemia (CML) on the translocated BCR-ABL kinase is correlated with dramatic responses to small molecule inhibitors. A large number of diverse point mutations that impair the binding of Imatinib (Gleevec) to ABL have been described [39]–[41], suggesting that some drug resistant mutations could exist before treatment, and may contribute to tumorigenesis. The profound selectivity of Imatinib at inhibiting a small group of protein tyrosine kinases is achieved by the high precision with which this inhibitor can recognize the inactive conformation of the activation loop in ABL, KIT and PDGFR kinases [42],[43]. Structurally conserved gate-keeper mutation ABL-T315I is a dominant cancer-causing alteration, leading to the most severe Imatinib resistance by favoring the active form of the ABL kinase. Subsequently, a series of rationally designed analogs of Imatinib based on the core scaffold were shown to recognize a broader spectrum of inactive kinase conformations and inhibit with equal potency both ABL and C-Src kinases [44]. Inhibitors that bind to the inactive conformation face weaker competition from cellular ATP and may act by shifting equilibrium between conformational states in a way that prevents kinase activation, rather than by inhibiting kinase activity directly.

A spectrum of lung cancer-derived EGFR mutations can induce oncogenic transformation by leading to constitutive kinase activity and confer markedly different degrees of sensitivity to EGFR inhibitors [45]–[47]. Similarly, EGFR-T790M mutant could cause resistance to Gefitinib and Erlotinib drugs in the treatment of lung cancer [48],. Importantly, these mutations can promote oncogenic activation, uncontrolled cell proliferation and tumorigenesis even in the absence of the selective pressure from the kinase inhibitors. An activating mutation in the activation loop of the EGFR kinase domain, L858R (also identified as Leu834 in a different numbering of the EGFR sequence) is among most frequent mutations in lung cancer, amounting to more than 40% of EGFR mutations in this cancer category [45]–[47]. While T790M has only a modest effect on EGFR function, a tandem of T790M and L858R mutations can result in a dramatic enhancement of EGFR activity [50]. The crystal structures of EGFR-L858R, EGFR-T790M [51]–[53] and ABL-T315I mutants [54],[55] have shown that these cancer-causing modifications could stabilize the active kinase form. Recent structural and mutagenesis investigations have asserted a common activating nature of the gatekeeper mutations in c-ABL, c-Src, and EGFR and PDGFR kinases [56]. Moreover, mutations of the gatekeeper residues to smaller amino acids and pharmacological intervention by the inhibitor binding, which interfere with the structural integrity of the hydrophobic spine, could effectively abrogate the kinase activity. Conversely, substitutions of the gatekeeper residues with bulkier modifications, that strengthen the hydrophobic spine, tend to correlate with the enhanced oncogenic activation of ABL and EGFR kinases [56]. These studies have proposed a mechanism of activation in which stabilization of the hydrophobic regulatory spine may promote shift of the kinase equilibrium towards the constitutively active kinase form, and thus have a dramatic effect on the regulation of the enzyme.

Crystallographic analysis may not capture the complete ensemble of protein kinase conformations available in solution under physiological conditions. NMR spectroscopy techniques can effectively complement X-ray studies by providing a more adequate characterization of conformational ensembles and dynamics of transitions between different kinase states [57],[58]. The first NMR characterization of ABL kinase in complexes with various inhibitors has been recently reported [57]. This study has detected microsecond to millisecond motions of the activation loop seen in both the active and inactive states, suggesting that this mobility may be an intrinsic structural requirement for enabling conformational transitions between alternative kinase conformations. Hydrogen exchange mass spectrometry (HX MS) has been applied to investigate conformational dynamics of ABL upon T315I mutation [58]. The effect of ABL-T315I mutation manifested not only in the local conformational disturbances near site of mutation, but also influenced protein flexibility in remote regions of the SH3 domain. Hence, allosteric interactions and inter-domain communication of ABL regulatory complexes could be considerably perturbed by activating mutations, thereby playing a major role in the kinase regulation in solution.

Computational studies have begun to investigate a molecular basis of protein kinase function and the structural effects of activating mutations, which may ultimately control the activity signatures of cancer drugs and determine the scope of drug resistance mutations [59],[60]. A molecular mechanism of long-range, allosteric conformational activation of Src tyrosine kinases has been proposed by using a combination of experimental enzyme kinetics and nonequilibrium molecular dynamics simulations [61],[62]. Atomistic simulations of large-scale allosteric conformational transitions of adenylate kinase have suggested a population-shift mechanism upon inhibitor binding [63]. Coarse-grained and all-toms modeling using structural connectivity mapping have allowed to characterize a collective dynamics of conformational transitions between the inactive and active states of the Src kinase [64]–[67]. Atomistic dynamics of the “open-to-closed” movement of the cyclin-dependent kinase 5 (CDK5) has been recently studied using a metadynamics sampling approach, revealing a two-step molecular mechanism and the formation of functionally important intermediates [68]. Molecular dynamics (MD) simulations of ABL kinase and Imatinib-binding kinetics assays have proposed that a protonation-dependent switch in the DFG motif from the activation loop may allow the kinase to access multiple conformations facilitating nucleotide binding and release cycles [69]. Targeted molecular dynamics (TMD) simulations have attempted to explore conformational transitions in the activation loop of the c-Kit kinase domain [70]. Most recently, conformational dynamics of the EGFR kinase domain studied by TMD simulations has suggested that formation of the hydrophobic spine and salt bridges may be important in the activation process [71]. Computational studies of protein kinases have elucidated thermodynamic factors of kinase activation, suggesting that cancer mutations with the higher oncogenic activity may have the greater destabilization effect on the inactive kinase structure [72],[73].

These studies have suggested that the conserved topology of the protein kinase fold could preserve global dynamics in the normal and oncogenic forms, yet allowing for functionally important local and allosteric conformational changes caused by mutations. The basic mechanistic features of the protein kinase dynamics and activation mechanisms may be interpreted using a conformational selection model [74]–[77] and the energy landscape perspective [74]–[80] of protein folding and binding. This theoretical framework implies an ensemble of preexisting multiple conformational states on the underlying energy landscape, with the mutations (or binding partners) shifting the energy landscape and the relative populations of accessible states towards functionally relevant complexes [74]–[77]. An important role of conformational selection mechanisms has recently gained further prominence [81], suggesting a broad applicability of this model in explaining dynamic effects for a variety of biological systems [82]–[85].

It was recently proposed that evolution may have preserved protein flexibility features that retain the ability of kinases to fluctuate normally between active and inactive states. In contrary, cancer kinase mutations may result in the increased conformational space to be explored in the inactive state [72],[73]. Thermodynamic and mechanistic effects of cancer mutations may manifest in a preferential shifting of the landscape equilibrium and altering of the accessible conformational space for deleterious mutants through either local or allosteric-based dynamic changes. A similar energy landscape-based framework for predicting the effects of mutations on protein dynamics and binding was successfully employed for allostery-based rescue mutant design in a tumor suppressor protein [86] and studies of molecular evolution of affinity and flexibility in the immune system [87],[88].

Despite recent progress in computational and experimental studies of protein kinases, a quantitative understanding of thermodynamic and mechanistic catalysts of kinase activation by cancer mutations is still lacking. In this study, we have embarked on a detailed computational analysis of activation mechanisms in the ABL and EGFR kinase domains using homology modeling, MD simulations, protein stability analysis, TMD simulations and molecular docking. A comparative analysis has been conducted based on computational modeling of the wild type (WT) ABL and EGFR kinase domains as well as a panel of clinically important cancer mutants ABL-T315I, ABL-L387M, EGFR-T790M, and EGFR-L858R. We have also simulated the effect of the gatekeeper ABL-T315I mutation on conformational dynamics and allosteric interactions in the ABL-SH2-SH3 regulatory complexes. In support of the experimental hypotheses, our results have suggested potential thermodynamic and mechanistic catalysts of the ABL and EGFR kinase activation that may collectively accelerate conformational transitions and result in the enhanced stabilization of the active kinase form. We have also proposed a multi-stage mechanistic model of the activation process that includes a series of cooperative transitions resulting in the formation of key intermediate states that are characterized by a rapid assembly of the hydrophobic spine and subsequent stabilization of the Src-like structures. Broadly, the results of study may reconcile current experimental data with the insights from computational approaches, pointing to general mechanistic aspects of activating transitions in protein kinases.

## Results/Discussion

Conformational landscapes of the ABL (**Figure S1**) and EGFR kinase domains (**Figure S2**) are characteristically marked by the presence of structurally different protein forms with a diverse arrangement of the activation loop seen in the inactive, Src-like inactive, and active states. A conserved K271-E286 salt bridge is present in the Imatinib-bound, inactive form **(Figure S1 D**) and the active form of ABL **(Figure S1 F**), while it is conspicuously absent in the Src-like inactive structure (**Figure S1 E**). The analogous conserved salt bridge K745-E762 is formed in the active EGFR conformation, but is dismantled in the Src/Cdk-like inactive conformation (**Figure S2 D–F**). The crystal structures of the ABL-T315I, EGFR-L858R, and EGFR-T790M mutants adopted the active kinase conformation. Moreover, a crystallographic analysis of EGFR-WT, EGFR-L858R, and EGFR-T790M indicated that activation of the kinase domain by cancer mutations should instigate a large conformational transition from the otherwise thermodynamically stable Src/Cdk-like inactive conformation [24]. Hence, structural effects of ABL and EGFR oncogenic mutations should lead to disruption of the autoinhibitory interactions in the inactive kinase conformation and promote a large conformational change of the activation loop.

### Homology Modeling of the ABL and EGFR Mutants

**Figure 1:**
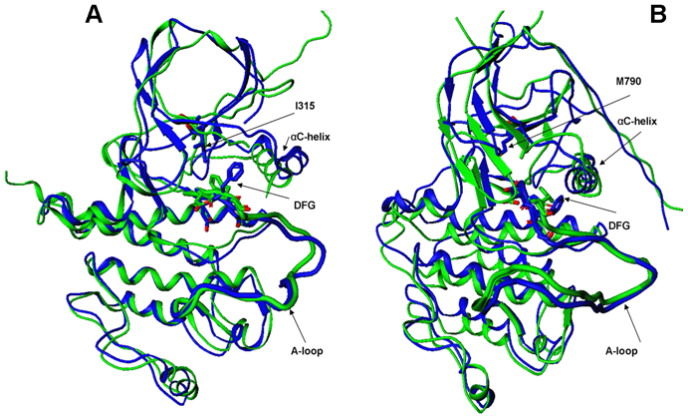
The predicted structural models of the ABL-T315I and EGFR-T790M mutants. (A) Superposition of the predicted structural model of the ABL-T315I mutant (in blue) with the crystal structure of ABL-T315I (active form, pdb entry 2Z60, in green). (B) Superposition of the predicted structural model of the EGFR-T790M mutant (in blue) with the crystal structure of EGFR-T790M (active form, pdb entry 2JIT, in green). The initial ABL and EGFR structures that converged during homology modeling refinement to the crystallographic active conformations of the mutants correspond to the Src-like inactive ABL (pdb entry 2G1T) and Src-like inactive EGFR (pdb entry 2GS7).

An important initial insight into structural effects of activating mutations was obtained from homology modeling of the conserved gatekeeper mutants ABL-T315I and EGFR-T790M. Structural analysis of ABL-T315I was attempted from the Imatinib-bound inactive and Src-like inactive conformations. Similarly, homology refinement of EGFR-T790M was carried out using the Src/Cdk-like inactive EGFR conformations. The predicted structural models conform closely to the crystallographic conformations of the activation loop in the mutant structures within the root mean square deviation (RMSD) of 2.0 Å–2.2 Å (**Figure 1**). It is worth noting that the target conformations of the activation loop in the mutant crystal structures assume the active form and were vastly different (RMSD ∼8 Å–10Å) from the starting conformations corresponding to the Src-like kinase state. Consequently, homology modeling of mutational effects in the ABL and EGFR kinase domains is rather challenging, albeit at a high sequence identity. While homology refinement could not accurately reproduce structural changes in the remote peripheral regions of the N-terminal and C-terminal lobes, simulations were capable of depicting the correct repositioning of the functionally important P-loop, αC-helix and the activation loop (**Figure 1**). A close structural inspection of the predicted ABL-T315I (**Figure S3A**) and EGFR-T790M models (**Figure S3B**) revealed a specific intermediate position of the Phe residue in the DFG motif that was favored by the enhanced hydrophobic interactions with the respective gatekeeper residue. Interestingly, while one copy of the EGFR-T790M crystal structure was in the active state with the characteristic “DFG-in” conformation, the second molecule displayed a rather unique intermediate DFG conformation [52]. The DFG conformation in the predicted model of EGFR-T790M closely overlapped with the one observed in the second molecule of the EGFR-T790M crystal structure. A convergent structural effect of the ABL-T315I and EGFR-T790M mutants (**Figure S3C**) seen in the predicted models may have functional relevance in facilitating conformational transition of the activation loop. The bulkier mutant residues at the gatekeeper position may unlock the DFG motif from its autoinhibitory conformation by strengthening the hydrophobic interactions with the Phe residue in the intermediate conformation. A specific DFG conformation, recruited by the mutated gatekeeper residues, may act as a conformational trigger in facilitating transition from the inactive DFG-out to the active DFG-in state. The proposed structural mechanism may thus initiate the disruption of the autoinhibitory interactions and cause imbalance in the dynamic equilibrium between inactive and active conformational states regulating kinase activity. Hence, homology modeling of the ABL-T315I and EGFR-T790M mutants could provide an initial insight into molecular basis of activation by cancer mutations.

**Figure 2:**
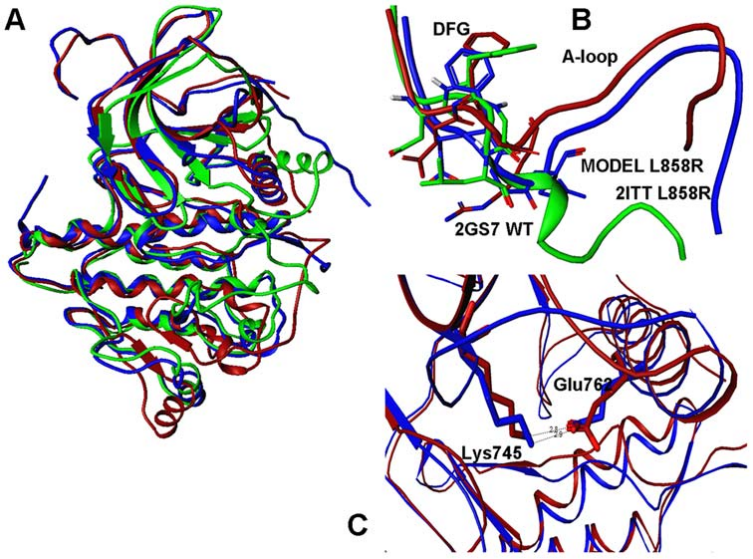
The predicted structural models and interactions of the EGFR-L858R mutant. (A) Superposition of the crystal structures for the inactive EGFR-WT structure (initial structure in homology refinement) (pdb entry 2GS7, in green), EGFR-L858R mutant crystal structure (target structure in homology refinement) (pdb entry 2ITT, in blue) and computationally predicted EGFR-L858R model (in red). (B) A close-up comparison between activation loop conformations in the crystal structures of inactive EGFR-WT (pdb entry 2J6M, in green), EGFR-L858R mutant crystal structure (pdb entry 2ITT, in blue) and the predicted mutant conformation (in red). The lowest energy mutant model is within RMSD = 1.98 Å from the crystal structure of EGFR-L858R. (C) A close-up of functionally important residues and key interactions stabilizing the active conformation of EGFR-L858R. The ion pairs between Lys-745 and Glu-762 are shown for the crystal structure of the EGFR-L8585R (in blue) and the predicted mutant conformation (in red).

Homology refinement successfully reconstructed the active crystallographic form of EGFR-L858R (pdb entry 2ITT) (**Figure 2A**). A considerable conformational rearrangement in the activation loop was predicted within RMSD = 1.9 Å–2.0 Å from the mutant crystal structure (**Figure 2B**). Moreover, homology refinement correctly reproduced coordinated structural changes of the activation loop and αC-helix (**Figure 2A**). The ability of homology modeling refinement to reproduce a large conformational change in EGFR-L858R may be partly attributed to a considerable incompatibility of Arg858 with the Src/Cdk-like inactive EGFR structure. In agreement with the assertion originally made by Kuriyan and coworkers [24], a local hydrophobic environment seemed to disfavor a polar side-chain of EGFR-L858R in the Src/Cdk-like inactive structure, and this may have facilitated a transition to a different local minimum corresponding to the active kinase form. A close inspection of the predicted structural model indicated that important interactions formed in the crystal structure of EGFR-L858R could be adequately reproduced, including a stable K745-E762 ion pair, known as a critical attribute of the active kinase form (**Figure 2C**).

### MD Simulations of the ABL/EGFR Kinase Domains and Protein Stability Analysis

**Figure 3:**
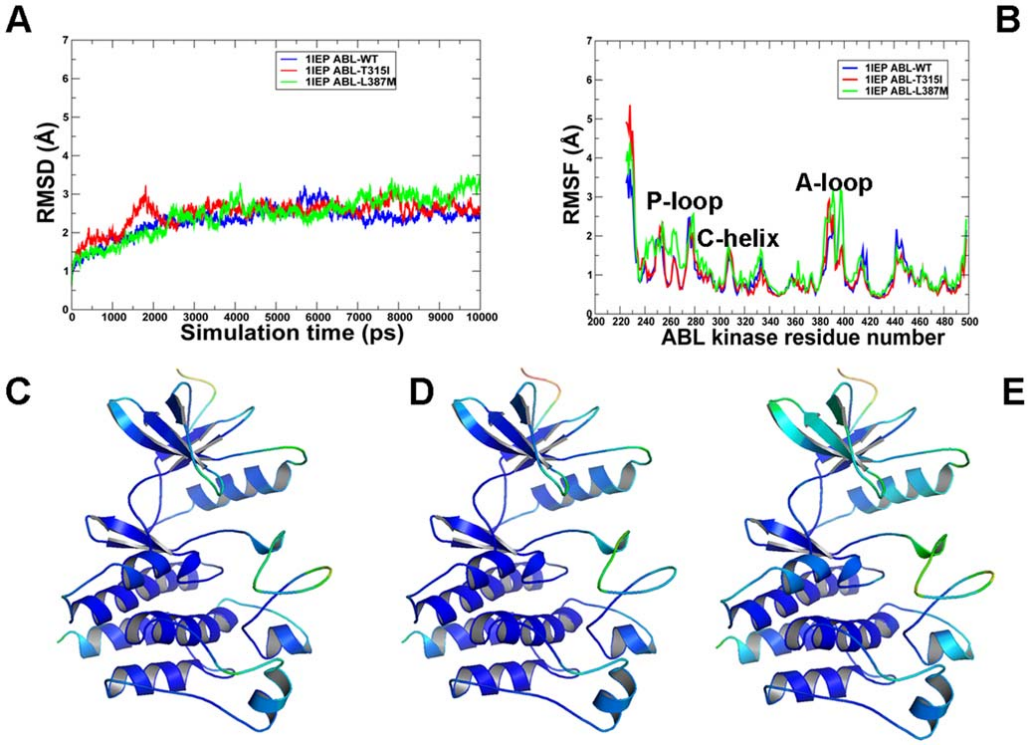
MD simulations of the ABL kinase domain in the imatinib-bound inactive form. Upper Panel: (A) The RMSD fluctuations of Cα atoms and (B) the RMSF values of Cα atoms from MD simulations of ABL-WT (in blue), ABL-T315I (in red), and ABL-L387M (in green). MD simulations were performed using the Imatinib-bound, inactive form of the ABL kinase domain. Legends inside the figure panels refer to the pdb entries used in MD simulations. The color-coded sliding scheme corresponds to the following ranges of protein flexibility values: red (highly flexible with +5.00 values), brown (+4.00 values), yellow (+3.00), green (+2.00), cyan (+1.00) and blue (the least flexible). Lower Panel: Color-coded mapping of the averaged protein flexibility profiles (RMSF values) from MD simulations of the ABL-WT and ABL mutants. This mapping is presented for ABL-WT (C), ABL-T315I (D) and ABL-L387M (E). The color-coded sliding scheme corresponds to the following ranges of protein flexibility values: red (highly flexible with +5.00 values), brown (+4.00 values), yellow (+3.00), green (+2.00), cyan (+1.00) and blue (the least flexible).

**Figure 4 pcbi-1000487-g004:**
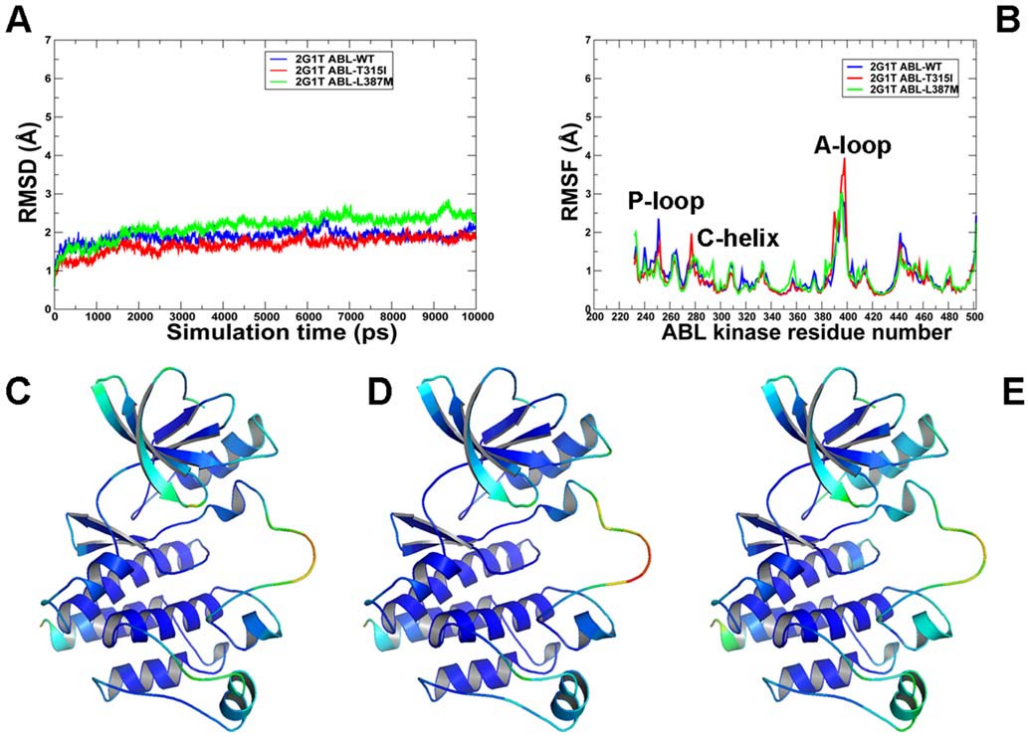
MD simulations of the ABL kinase domain in the Src-like inactive form. Upper Panel: (A) The RMSD fluctuations of Cα atoms and (B) the RMSF values of Cα atoms from MD simulations of ABL-WT (in blue), ABL-T315I (in red), and ABL-L387M (in green). MD simulations were performed using the Src-like inactive form of the ABL kinase domain. Legends inside the figure panels refer to the pdb entries used in MD simulations. Lower Panel: Color-coded mapping of the averaged protein flexibility profiles (RMSF values) from MD simulations of the ABL-WT and ABL mutants. This mapping is presented for ABL-WT (C), ABL-T315I (D) and ABL-L387M (E). The color-coded sliding scheme is the same as was adopted for Figure 3.

MD simulations of the ABL and EGFR kinase domains were performed to address the following objectives: (a) to determine whether thermal motions in structurally different conformational states of ABL and EGFR may tolerate structural impact of activating mutations; (b) to study equilibrium dynamics of the ABL and EGFR kinase domain in the normal and oncogenic forms; and (c) to investigate the impact of activating mutations on the thermodynamic stability of structurally different conformational states of ABL and EGFR. MD trajectories of the ABL kinase domain (**Figures 3** **–** [Fig pcbi-1000487-g004] **5**) and EGFR kinase domain (**Figures 6** **,** **7**) displayed important similarities and differences in the equilibrium profile of the WT and mutant forms. The RMSD fluctuations of the Src-like ABL (**Figures 4**) and Src/Cdk-like EGFR (**Figure 6**) displayed a similar convergence to an equilibrium plateau within first 2 ns–3 ns and remained stable for the remainder of simulations. Characteristically, mutations can induce larger thermal fluctuations in the inactive kinase state (RMSD = 2.2 Å–2.5 Å), especially for ABL-L387M and EGFR-L858R. Overall, there were no significant differences between the RMSD profiles of ABL and EGFR in structurally different kinase states, although thermal fluctuations of the EGFR variants were somewhat smaller (1.5Å–2.0Å) (**Figures 6** **,** **7**). We also analyzed protein flexibility variations computed from the root mean square fluctuation (RMSF) of the backbone residues. To facilitate the analysis, the average protein flexibility profiles were mapped onto the respective conformational states of ABL and EGFR using a color-coded sliding scheme. In agreement with the structural factors, the regions of larger thermal fluctuations and the increased conformational flexibility corresponded to P-loop, αC-helix and the activation loop. During simulations in the Src-like inactive states, we observed a consistently increased level of thermal fluctuations for the ABL-L387M **(** **Figure 4**) and EGFR-L858R mutants (**Figure 6**) that are strategically located in the middle of the activation loop and may thus cause larger local perturbations. In contrast, EGFR-L858R mutant displayed the decreased thermal fluctuations and smaller RMSF values in the active state (**Figure 7**). This may partly reflect the activating nature of the EGFR-L858R mutation that is known to shift the thermodynamic equilibrium away from the Src/Cdk-like form towards a more stable active kinase structure.

**Figure 5:**
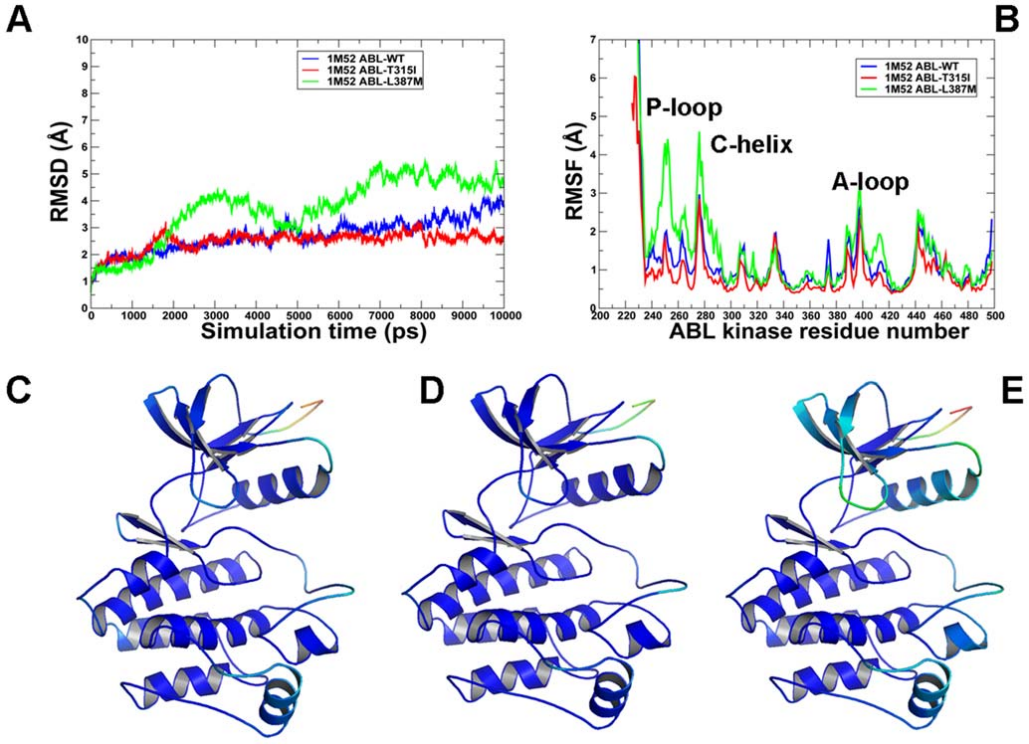
MD simulations of the ABL kinase domain in the active form. Upper Panel: (A) The RMSD fluctuations of Cα atoms and (B) the RMSF values of Cα atoms from MD simulations of ABL-WT (in blue), ABL-T315I (in red), and ABL-L387M (in green). MD simulations were performed using the active form of the ABL kinase domain. Legends inside the figure panels refer to the pdb entries used in MD simulations. Lower Panel: Color-coded mapping of the averaged protein flexibility profiles (RMSF values) from MD simulations of the ABL-WT and ABL mutants. This mapping is presented for ABL-WT (C), ABL-T315I (D) and ABL-L387M (E). The color-coded sliding scheme is the same as was adopted for Figure 3.

**Figure 6:**
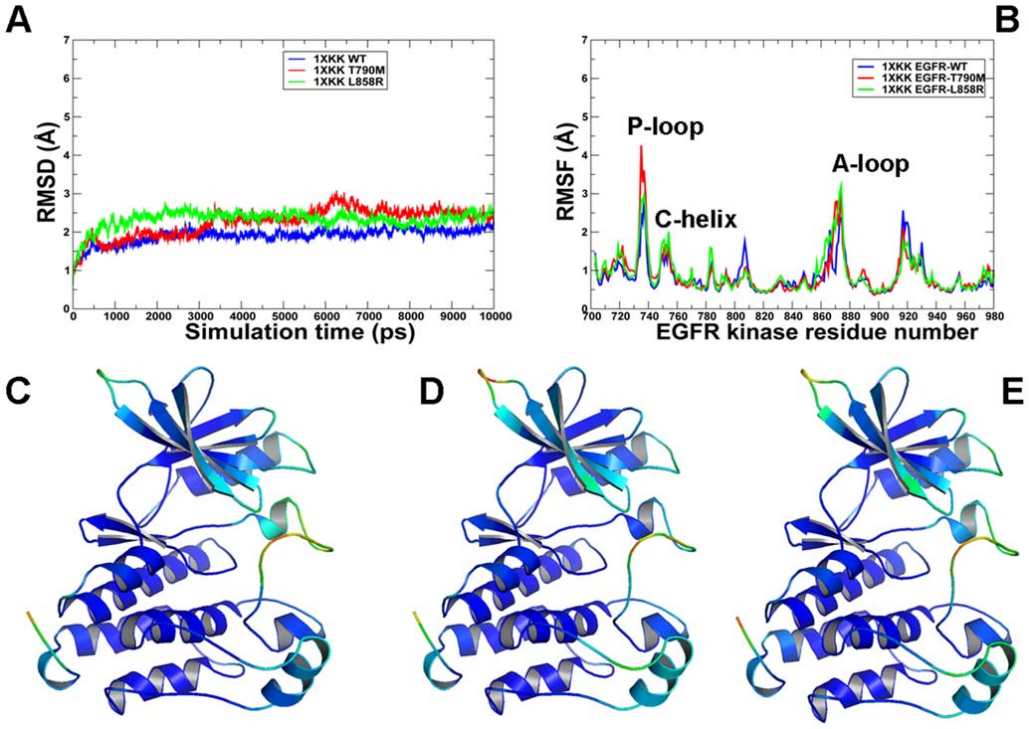
MD simulations of the EGFR kinase domain in the Src/Cdk-like inactive form. Upper Panel: (A) The RMSD fluctuations of Cα atoms and (B) the RMSF values of Cα atoms from MD simulations of EGFR-WT (in blue), EGFR-T790M (in red), and EGFR-L858R (in green). MD simulations were performed using the Src/Cdk-like inactive form of the EGFR kinase domain. Legends inside the figure panels refer to the pdb entries used in MD simulations. Lower panel: Color-coded mapping of the averaged protein flexibility profiles (RMSF values) from MD simulations of the EGFR-WT and EGFR mutants. This mapping is presented for EGFR-WT (C), EGFR-T790M (D) and EGFR-L858R (E). The color-coded sliding scheme is the same as was adopted for Figure 3.

**Figure 7:**
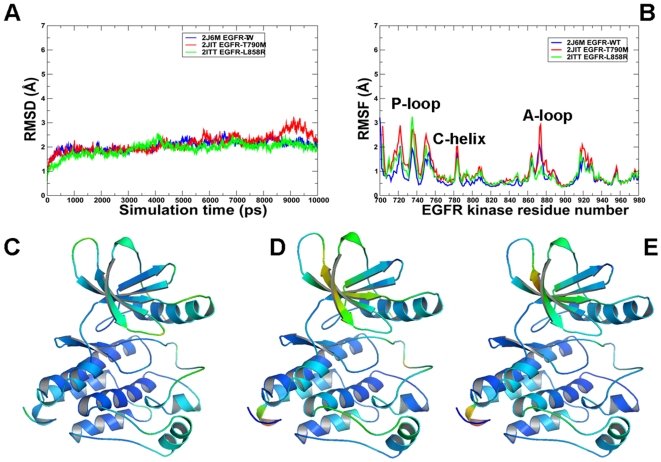
MD simulations of the EGFR kinase domain in the active form. Upper Panel: (A) The RMSD fluctuations of Cα atoms and (B) the RMSF values of Cα atoms from MD simulations of EGFR-WT (in blue), EGFR-T790M (in red), and EGFR-L858R (in green). MD simulations were performed using the active form of the EGFR kinase domain. Legends inside the figure panels refer to the pdb entries used in MD simulations. Lower Panel: Color-coded mapping of the averaged protein flexibility profiles (RMSF values) from MD simulations of the EGFR-WT and EGFR mutants. This mapping is presented for EGFR-WT (C), EGFR-T790M (D) and EGFR-L858R (E). The color-coded sliding scheme is the same as was adopted for Figure 3.

**Figure 8:**
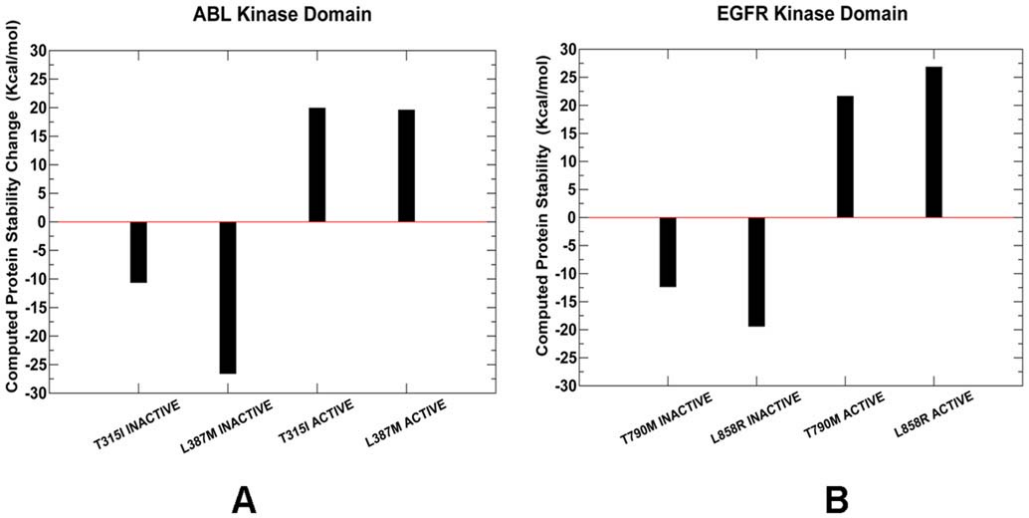
The protein stability analysis of the ABL and EGFR cancer mutants. Left Panel (A): The computed protein stability changes between ABL-WT and ABL-T315I, ABL-L387M mutants in the Src-like inactive and active states of ABL. Right Panel (B): The computed protein stability changes upon EGFR-T790M, EGFR-L858R mutants in the Src/Cdk-like inactive and active states of EGFR. In both panels, a negative value in the protein stability difference between the WT kinase and a respective mutant corresponds to a destabilization effect of the mutant.

Analysis of the time-dependent RMSD and RMSF fluctuations could provide only an initial and largely qualitative outlook of potential protein stability changes that may be induced by mutations. The thermodynamic effect of activating mutations could be quantified more rigorously when the results of equilibrium MD simulations are combined with the MM-GBSA analysis of protein stability change (**Figure 8**). Coupling between rigid and flexible regions of a protein and correlation of various motions may generally lead to both increases and decreases in thermodynamic stability. Our analysis demonstrated a moderate decrease in the thermodynamic stability for ABL-T315I and a more significant effect of ABL-L387M on the Src-like, inactive structure (**Table 1**). The detrimental energetic effect of ABL-T315I on the inactive structure was due to loss of the molecular mechanics energy, and specifically its electrostatic component. The ABL-L387M mutation could lead to a considerable decrease in the favorable van der Waals interactions as well as concurrent unfavorable changes in solvation free energy (**Table 1** **,** **Figure 8A**). In contrast, both activating mutations may appreciably stabilize the active form of ABL (**Table 1**). All free energy components appeared to act concertedly to enhance the thermodynamic stability of the active form. In particular, the van der Waals interactions were strengthened in the active form of ABL-T315I, partly owing to a favorable packing of I315 with the Met290 and Leu301 residues from the hydrophobic spine. The results of simulations provided an interesting rationale to the previously proposed structural effect of the L387M mutation on kinase activity [18]. This hydrophobic residue is solvent-exposed in both inactive and active forms of ABL, while it faces the interface between αC-helix, the N-lobe, and the activation loop in the Src-like inactive structure (**Figure S1**). According to the original conjecture by Kuriyan and coworkers, functional effect of the activating ABL-L387M may be explained if the Src-like inactive conformation could represent a significant population of the inactive ABL states during thermodynamic equilibrium [18]. Our simulations allowed a quantitative insight that supported this hypothesis. Indeed, local adjustments of the activation loop at the mutational site may not rescue the weakening packing interactions caused by L387M in the Src-like structure, and thus could lead to an appreciable decrease in the thermodynamic stability. At the same time, ABL-L387M enhanced the thermodynamic stability of the active kinase state (**Table 1** **,** **Figure 8A**).

**Table 1:** MM-GBSA calculations of the protein stability for the ABL kinase*.

Energy	WT(I)	T315I(I)	L387M(I)	WT(A)	T315I(A)	L387M(A)
**E_ele_**	−7874.34	−7839.11	−7935.50	−7940.54	−7948.96	−7880.00
**E_vdw_**	−1595.93	−1592.86	−1560.10	−1548.94	−1568.18	−1559.64
**E_int_**	3028.50	3032.25	3045.91	3040.95	3043.89	3037.51
**E_gas_**	−6441.77	−6399.72	−6449.69	−6448.44	−6473.25	−6402.13
**G_gbnb_**	102.98	104.05	99.95	103.75	100.10	102.57
**G_gbele_**	−3409.06	−3435.75	−3369.63	−3389.31	−3383.73	−3460.25
**G_gbtot_**	−3306.07	−3331.70	−3269.69	−3285.57	−3283.63	−3357.68
**TS_trans_**	17.06	17.06	17.06	17.06	17.06	17.06
**TS_rot_**	17.61	17.61	17.61	17.60	17.57	17.59
**TS_vib_**	3167.18	3173.00	3169.09	3166.58	3163.65	3160.37
**T_Stotal_**	3201.86	3207.67	3203.76	3201.24	3198.29	3195.03

*: The columns 2–4 represent the protein stability contributions calculated for the Src-like inactive form of ABL for the WT, T3135I and L387M mutants. The columns 5–7 represent the respective protein stability contributions calculated in the active form of ABL for the WT, T315I and L387M mutants. All energies are in Kcal/mol. The contributions of the free energies are defined in the Materials and Methods section.

Similar effects could be observed from the protein stability analysis of EGFR mutations (**Table 2** **,** **Figure 8B**). In this case, both EGFR-T790M and EGFR-L858R induced a detrimental effect on the Src/Cdk-like inactive state through the decreased electrostatic and van der Waals interactions, which could not be offset by the opposing changes in solvation free energy (**Table 2**). A greater local mobility induced by mutations in the Src-like inactive structure was reflected in rather moderate net contributions of the vibrational entropy and solvation energy. Importantly, we found that a more significant loss of thermodynamic stability in the Src-like inactive form may be caused by ABL-L387M and EGFR-L858R mutations in the activation loop (**Tables 1** **,** **2**). In contrast, both EGFR mutants may lead to the enhanced thermodynamic stability of the active structure. The crystal structure analysis previously indicated that EGFR-L858R may be incompatible with the local hydrophobic environment in the Src/Cdk inactive structure [24]. In agreement with the structural data and the original conjecture proposed by Kuriyan and colleagues, we determined that mutations with the higher oncogenic activity, such as a frequently occurring in lung cancer EGFR-L858R, may induce the greater differential effect on thermodynamic stability of the inactive and active kinase forms. We found that the thermodynamic effect of activating mutations in ABL and EGFR may lead to simultaneous destabilization of the inactive kinase and the enhanced stability of the active state. Collectively, these energetic factors may serve as thermodynamic catalysts of kinase activation by cancer mutations that would favor shift in the equilibrium towards the active kinase form. The computed protein stability changes, caused by cancer mutations, typically tend to overestimate the absolute value of thermodynamic variations, due to the inherent nature of large compensating contributions involved in the MM-GBSA analysis. Nevertheless, our analysis allowed to reconcile the experimental data and quantified the differential thermodynamic effect of mutations on the inactive and active kinase forms.

**Table 2:** MM-GBSA calculations of the protein stability for the EGFR kinase*.

Energy	WT(I)	T790M(I)	L858R(I)	WT(A)	T790M(A)	L858R(A)
**E_ele_**	−6408.32	−6310.38	−6351.15	−6502.31	−6556.92	−6548.48
**E_vdw_**	−1687.45	−1641.53	−1657.98	−1712.21	−1679.19	−1670.60
**E_int_**	3172.4	3152.16	3159.98	3135.95	3146.32	3162.52
**E_gas_**	−4923.32	−4799.76	−4849.15	−5078.57	−5089.79	−5056.56
**G_gbnb_**	105.03	101.13	98.60	115.97	101.66	97.58
**G_gbele_**	−3531.67	−3638.02	−3576.44	−3547.51	−3545.82	−3572.07
**G_gbtot_**	−3426.64	−3536.89	−3477.83	−3431.54	−3444.16	−3474.49
**TS_trans_**	17.07	17.07	17.07	17.14	17.07	17.07
**TS_rot_**	17.59	17.58	17.58	17.75	17.59	17.59
**TS_vib_**	3204.19	3205.19	3207.81	3226.80	3224.80	3232.91
**T_Stotal_**	3238.85	3239.84	3242.45	3261.68	3259.46	3267.57

*: The columns 2–4 represent the protein stability contributions calculated for the Src/Cdk-like inactive form of EGFR for the WT, T790M and L858R mutants. The columns 5–7 represent the respective protein stability contributions calculated in the active form of EGFR for the WT, T790M and 858R mutants. All energies are in Kcal/mol. The contributions of the free energies are defined in the Materials and Methods section.

It is worth noting that activating mutations may induce not only local protein adjustments near the mutational site, but also cause allosteric changes in the αC-helix and N-terminal lobe. However, conformational plasticity of the kinase domain allowed to preserve structural integrity of the kinase conformational forms. These results agreed with the NMR studies, in which inactive ABL conformations were found to exhibit nanosecond backbone flexibility within the activation loop, yet the conformational ensemble on average could still closely conform to the crystal structure [57]. To probe a possibility of observing direct conformational transitions between the inactive and active kinase forms, we extended the time scale of MD simulations, but the observed profile of thermal fluctuations remained qualitatively the same. The recent NMR analysis [57] indicated that the microsecond to millisecond time scales associated with the activation loop motions may enable conformational transitions between alternative kinase conformations. Hence, computationally feasible time scales of MD simulations may not be sufficient to directly observe functionally important conformational transitions between structurally different kinase forms.

### MD Simulations of the ABL and EGFR Regulatory Complexes

**Figure 9:**
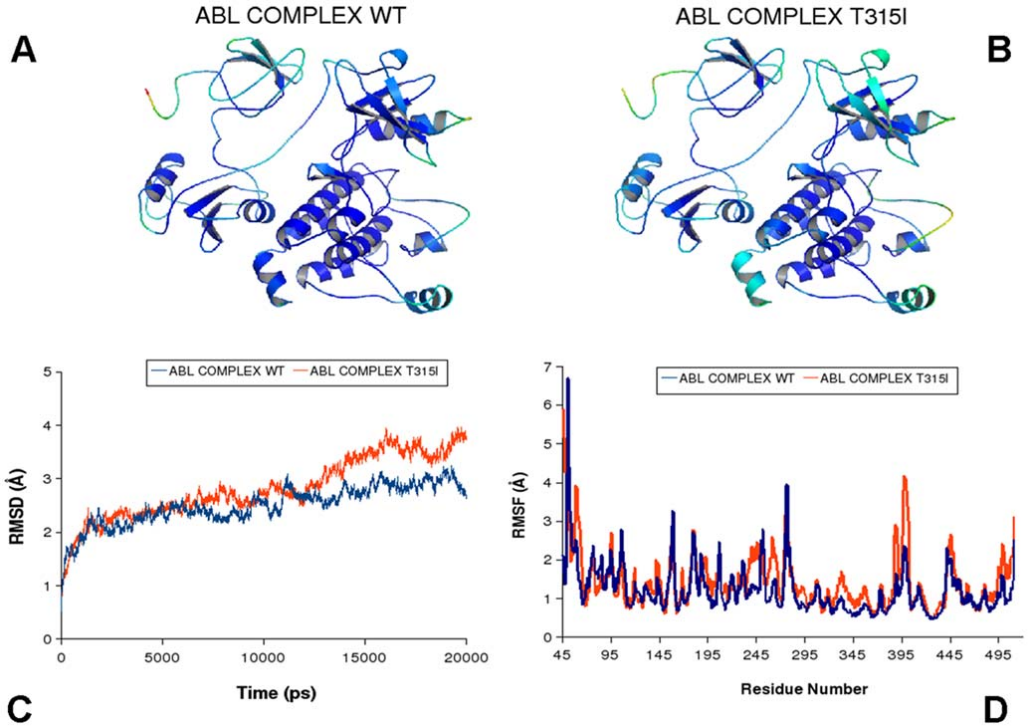
MD simulations of the ABL-SH2-SH3 regulatory complex in the inactive Form. Upper Panel: Color-coded mapping of the averaged protein flexibility profiles (RMSF values) from MD simulations in the inactive form (pdb entry 2FO0). The mapping is presented for ABL-WT (A) and ABL-T315I (B). The color-coded sliding scheme is the same as was adopted for Figure 3. Lower Panel: The RMSD fluctuations of Cα Atoms (C) and the RMSF values of Cα Atoms (D) from MD simulations. MD simulations of ABL-WT (in blue), and ABL-T315I (in red) were performed using the downregulated inactive ABL form (“side-to-side”) (pdb entry 2FO0).

**Figure 10:**
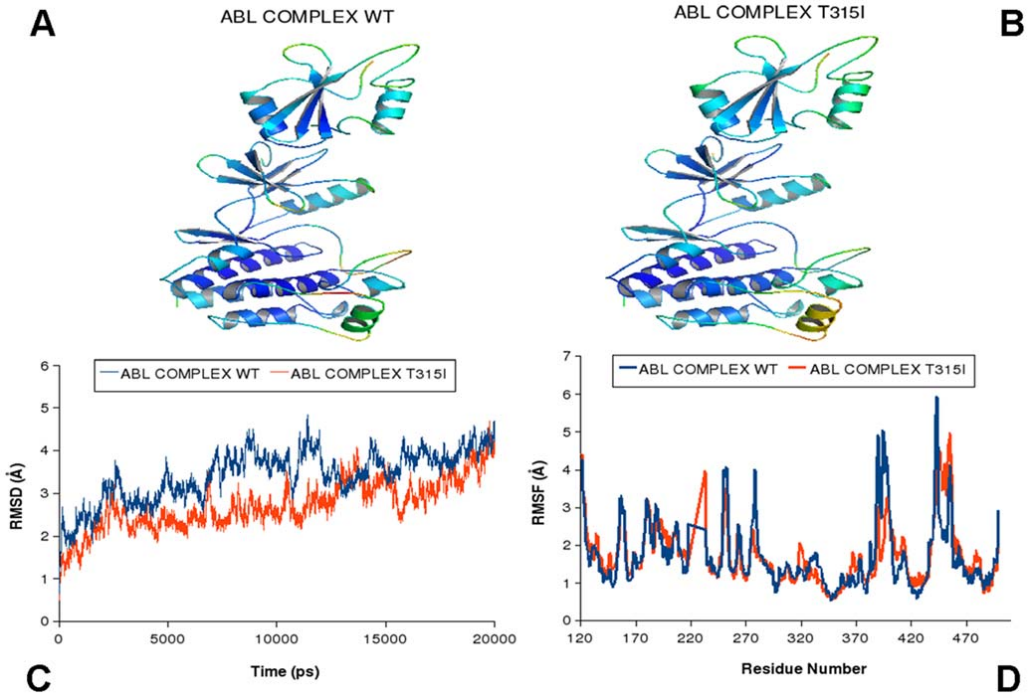
MD simulations of the ABL-SH2-SH3 regulatory complex in the active form. Upper Panel: Color-coded mapping of the averaged protein flexibility profiles (RMSF values) from MD simulations in the active form (pdb entry 1OPL). The mapping is presented for ABL-WT (A) and ABL-T315I (B). The color-coded sliding scheme is the same as was adopted for Figure 3. Lower Panel: The RMSD fluctuations of Cα Atoms (C) and the RMSF values of Cα Atoms (D) from MD simulations. MD simulations of ABL-WT (in blue), and ABL-T315I (in red) were performed using the active ABL form (“top-hat”) (pdb entry 1OPL).

Although ABL and EGFR share a common kinase domain, they can be regulated through different mechanisms. Indeed, activation of the ABL kinases can be linked with the formation of multi-protein regulatory complexes with the SH2 and SH3 domains [13],[17], whereas EGFR may be activated through dimerization mechanism [24]. These regulatory interactions have a major role in determining the conformational dynamics of the kinase domain and activation mechanisms. Crystallographic studies [13],[17] demonstrated that the ABL regulatory complex in the downregulated, inactive can form a tightly assembled conformation in which the SH3-SH2 unit docked onto the back of the kinase domain, considerably restricting its conformational flexibility (**Figure 9**). Moreover, a critical N-terminal cap segment connecting the myristoyl group to the SH3 domain contributed to the stability of the inactive ABL state (pdb entry 2FO0) [89],[90]. Small angle X-ray scattering (SAXS) analysis of the activated ABL form revealed that the release of the inhibitory interactions could allow the SH2 and SH3 domains to switch to a vastly different structural arrangement [17]. In this structure (**Figure 10**), the SH2 domain was docked directly on the top of the N-lobe (“top-hat” conformation) (pdb entry 1OPL), while the SH3 domain became disordered along with the linker connecting the SH2 and the kinase domains. To gain dynamic and mechanistic insight into allosteric effects of the ABL-T315I mutant, we performed MD simulations of the ABL-SH2-SH3 regulatory complexes in the inactive (“side-to-side”) and active (“top-hat”) states. The objectives of these simulations were the following: (a) to determine the effect of activated ABL-T315I mutation on the conformational dynamics in different functional states of ABL; (b) to test a hypothesis whether upregulation of kinase activity, caused by ABL-T315I, may be related to the dynamic preferences in the “top-hat” conformation of the ABL-SH2-SH3 complex. The results of simulations were analyzed using a direct comparison with the recent HX MS experiments of ABL regulatory complexes in the normal and mutational forms [58].

A distinct pattern of structural flexibility upon T315I mutation was found from simulations in the inactive ABL state (**Figure 9**). The ABL-T315I mutant exhibited a considerably higher degree of thermal fluctuations as compared to the ABL-WT. The RMSD profile of ABL-WT in this downregulated form displayed minor deviations of ∼2Å for most of the 20 ns trajectory, suggesting that ABL-WT could be dynamically very stable in the inactive form. In contrast, ABL-T315I caused the increasing thermal perturbations on a longer time scale (after first 12 ns), suggesting a considerable structural rearrangement of the regulatory complex upon activating mutation. The RMSF profile revealed the regions of higher flexibility, including the RT-loop (residues 89–107), αC-helix (residues 281–291), activation loop (especially in the region of residues 383–402), and the α-helix at C-terminus (residues 502–512), the linker region (residues 209–237) and the N-cap (residues 45–66). Our results demonstrated an excellent agreement with the HX-MS experiments [58], since precisely these structural regions exhibited a higher exposure to solvent and conformational mobility evidenced by higher deuterium exchange. Also in agreement with the experiments, the least mobile regions were the bundle of α-helices in the large lobe of the ABL kinase domain and the SH2 domain itself (**Figure 9**).

Importantly, we detected subtle, yet functionally relevant dynamic changes in residues 255–275 and 285–300 (near ATP binding site) as well as allosteric effects that could occur in the distant RT-loop of the SH3 domain (residues 89–107). The RMSF profiles of ABL-T315I showed the increased conformational fluctuations of these regions in the inactive state (**Figure 9**). In agreement with the experiment, the altered dynamics of residues 287–302 can impact the stability of the hydrophobic spine. Hence, this local effect might potentially trigger a shift in equilibrium towards the active, “top-hat” conformation of the regulatory complex. Of particular importance were also the observed dynamic changes at the allosteric site of the RT-loop (**Figure 9**). In accordance with the experimental analysis, this region could be less structurally organized in ABL-T315I, whereby altered dynamics of the regulatory SH3 domain may ultimately lead to the increased kinase activity of the mutant. In contrast, simulations of the ABL complexes in the active, ”top-hat” form showed a reverse trend, i.e. the increased thermal fluctuations and the increase flexibility of ABL-WT as compared to the ABL-T315I mutant (**Figure 10**). Indeed, mutant complex revealed considerably smaller protein fluctuations in functionally important regions, especially in the activation loop region (residues 380–400) and in the ATP binding region (**Figure 10**). Hence, the activating mutation may shift dynamic preferences of the regulatory complex towards the active conformation. Collectively, these findings corroborated and reconciled the HX MS experimental data [58], indicating that a subtle impact of the ABL-T315I mutation could result not only in the localized conformational disturbances near the immediate site of mutation (region 287–302), but also influence protein flexibility in distant regions, including the SH3 domain (RT-loop residues 89–107). These dynamic alterations in the kinase domain and RT-loop may serve as important mechanistic triggers facilitating thermodynamic stabilization of the “top-hat” active state. Multiscale simulations of allosteric communications in the ABL regulatory complexes would be required to provide further insights into molecular basis of kinase activation.

Crystallographic and functional studies of EGFR activation mechanisms have shown that the EGFR kinase domain is intrinsically autoinhibited, and an intermolecular interaction in the asymmetric dimer can promote its activation [24]. In contrast, the symmetric dimer with two kinase domains interacting through a “head-to-tail” arrangement proved to be irrelevant for EGFR activation [24]. Recent MD simulations of the asymmetric EGFR dimer have confirmed a “positive” effect of activating mutations in stabilizing key structural elements associated with EGFR dimerization [71]. To complement this computational study, we sought to investigate a potential “negative” impact of the activating mutation on conformational dynamics in the symmetric dimer form. We proposed that the activating mutation may induce considerable perturbations in the symmetric dimer, since this structure appeared to be incompatible with the EGFR activation mechanism. Indeed, a comparison of the RMSD profiles indicated that EGFR-WT could become stable early (after ∼6 ns), followed by minor thermal fluctuations in a narrow range throughput the simulation period. In contrast, RMSD values for EGFR-T790M steadily increased in the course of simulations and beyond 20 ns (**Figure S4**). These results suggested that the activating mutation could induce a significant structural reorganization in the symmetric dimer, as this structure may not be important for the activation process. Analysis of the RMSF profiles further strengthened these arguments as EGFR-T790M showed a higher degree of structural fluctuation in the activation loop (residue 865–875). The second region of high conformational mobility corresponded to α-helix at the C-terminal part of the two monomers (residues 916–930), which was also more flexible in the mutant. A more systematic analysis of allosteric transitions during EGFR activation would require not only further computational efforts but also complementary structural and functional insights into the impact of specific activating mutations. This analysis extends beyond the scope of this manuscript and will be presented elsewhere.

### Targeted Molecular Dynamics Simulations of the ABL and EGFR Kinase Domains

In the current study, TMD simulations of the ABL and EGFR kinase domains were used symbiotically with other computational approaches to systematically address both thermodynamic and mechanistic aspects of kinase activation and compare these effects between normal and oncogenic kinase forms. We pursued the following specific objectives: (a) to determine whether conformational transitions of the activation loop could bring about collective motions in other functionally important kinase regions that may serve as mechanistic “wheels” of activation; (b) to determine similarities and differences between activation pathways in the ABL and EGFR kinase domains; (c) to propose a mechanistic model of the activation mechanism and determine molecular catalysts of ABL and EGFR activation.

**Figure 11:**
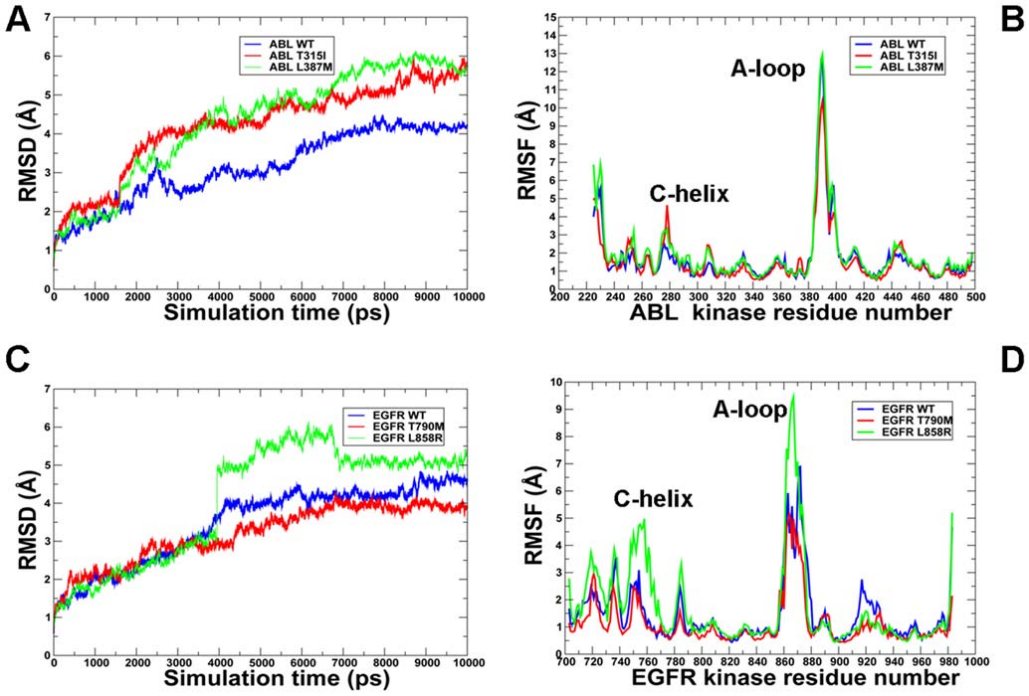
TMD simulations of the ABL and EGFR kinase domains. Time-dependent RMSD profile of Cα atoms obtained from TMD simulations of ABL (A) and EGFR kinase domains (C). The RMSF values of Cα atoms obtained from TMD simulations of ABL (B) and EGFR kinase domains (D). ABL-WT/EGFR-WT (in blue), ABL-T315I/EGFR-T790M (in red), and ABL-L387M/EGFR-L858R (in green). Legends inside the figure panels refer to the pdb entries corresponding to MD simulations from different conformational states of the kinase domain.

The RMSD profiles obtained from TMD simulations of the ABL (**Figure 11A**) and EGFR kinase domain (**Figure 11 C**) revealed characteristic trends in the nature of activation conformational transitions. In all cases, we observed a first transition, reflected in a rapid increase of RMSD values after 2–4 ns, gradual RMSD increase in the next 4–5 ns, followed by a second transition and attainment of the target active structure. The RMSD profiles of ABL-T315I and EGFR-T790M mutants exhibited a sharper first transition, relative to ABL-WT, followed by a second transition occurring at the onset of 4 ns–5 ns (**Figure 11 A,C**). More pronounced were the differences detected in the RMSD profile of the activation loop mutants ABL-L387 and EGFR-L858R, revealing characteristic “first-order-like” transitions (**Figure 11 A,C**). The RMSF profiles displayed a greater mobility of the P-loop, αC-helix and the activation loop as compared to other kinase regions (**Figure 11 B, D**). It is worth noting that the steering force was applied only to the activation loop residues, so that other kinase regions could move freely to accommodate to the progressive conformational changes. In general, RMSF fluctuations of the activation loop and αC-helix regions were similar between WT and mutant kinases, with the noticeable exception of EGFR-L858R revealing a considerably greater degree of mobility (**Figure 11 C,D**). Overall, the RMSF profiles showed appreciable αC-helix fluctuations (with RMSF >2Å) in response to the conformational changes in the activation loop, suggesting that collective motions of these kinase regions may play a mechanistic role in the activation process.

**Figure 12:**
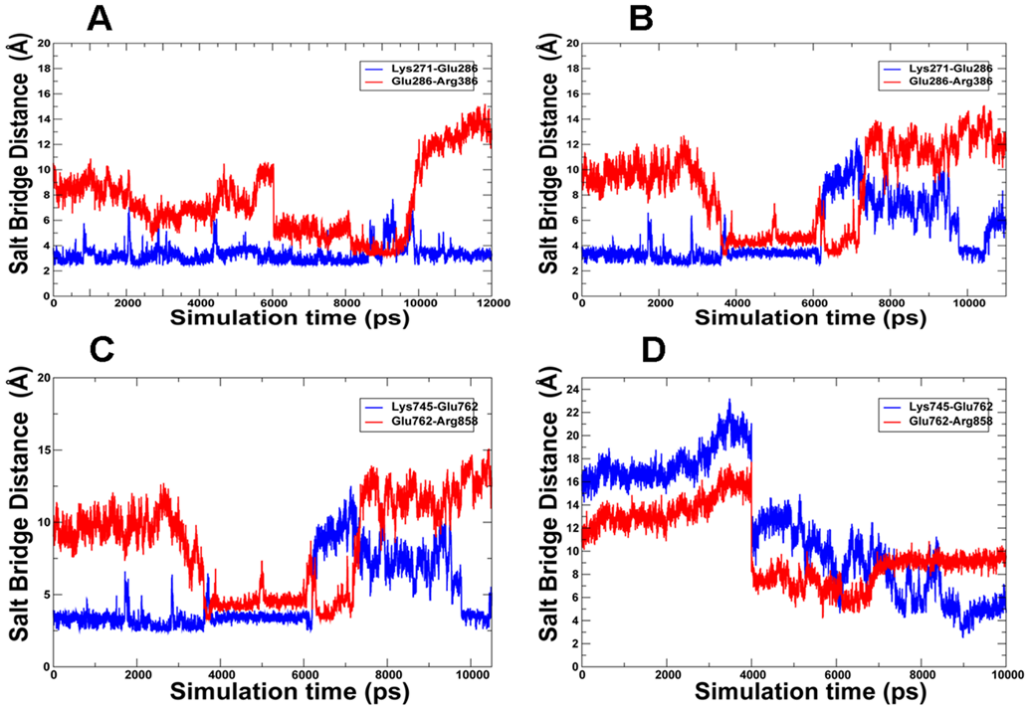
Time evolution of characteristic salt bridges in TMD simulations. The time evolution profiles of the formation and breakage of characteristic salt bridge profiles are shown for ABL-T315I (A), ABL-L387M (B), EGFR-T790M (C) and EGFR-L858R (D).

**Figure 13:**
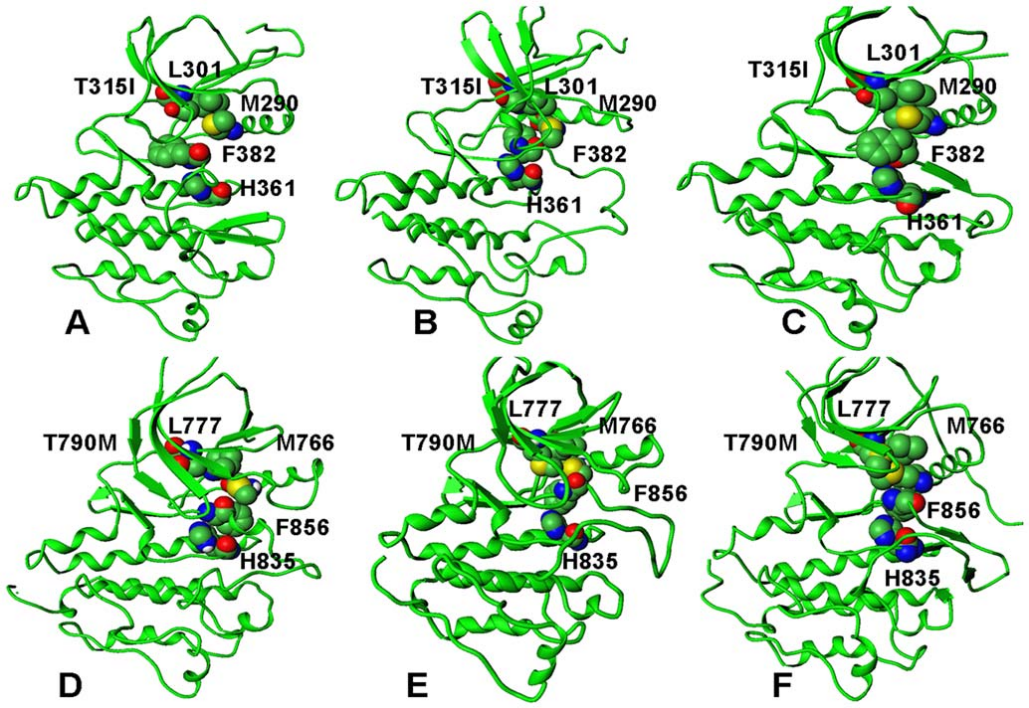
A mechanistic analysis of the TMD activation pathways for the ABL- T315I and EGFR-T790M gatekeeper mutations. A dynamic assembly of the hydrophobic spine interactions during the activation process is shown for the ABL-T315I mutant (upper panel) and the EGFR-T790M mutant (lower panel). The hydrophobic spine of the ABL kinase domain is formed through assembly of the gate-keeper residue with the Met290, Leu301, His361, and Phe382 residues. The hydrophobic spine of the EGFR kinase domain is similarly formed by linking the gate-keeper residue with Met766, Leu777, His835, and Phe856 residues respectively. (A) The initial model of ABL-T315I in the inactive form. (B) The characteristic TMD intermediate of ABL-T315I. (C) The final structure of ABL-T315I in the active form. (D) The initial model of EGFR-T790M in the inactive form. (B) The characteristic TMD intermediate of EGFR-T790M. (C) The final structure of EGFR-T790M in the active form. The assembled hydrophobic spine is present in all TMD intermediates. The determined meta-stable ABL intermediate closely resembles the Src-like inactive crystal structure.

Structural and energetic aspects of the activation process were analyzed by monitoring crucial structural elements of the activation process, i.e. the dynamic assembly of the hydrophobic spine and the formation of key salt bridge interactions important in the kinase regulation. The analysis of TMD simulations for ABL and EGFR mutants provided evidence of a common multi-stage activation process that could involve cooperative transitions between different conformational states of the kinase domain (**Figures 12**). Here, we detail the characteristic stages of the activation pathways by dissecting the results of TMD simulations for ABL mutants. At the first stage, modest fluctuations of the K271-E286 salt bridge, an important structural attribute of the Imatinib-bound ABL inactive state, were accompanied by a first sharp transition and an abrupt decrease of the E286-R386 distances (at the onset of ∼4 ns) (**Figure 12A,B**). The first transition could be determined by a rapid and cooperative assembly of the hydrophobic spine, which may be a common mechanistic event, shared by ABL and EGFR kinases (**Figures 13** **,** **14**). In particular, structural consolidation of the hydrophobic spine residues in ABL (Met290, Leu301, His361, and Phe382) may serve as a mechanistic “trigger” of the activation process to catalyze subsequent rearrangement of the K271-E286 and E286-R386 pairs in ABL-T315I and ABL-L387M mutants. The second transition could be prompted by the further consolidation of the hydrophobic spine, a concerted and sharp breakage of the K271-E286 salt bridge (loss of the Imatinib-bound inactive structure) and a concomitant formation of the E286-R386 ion pair (**Figure 12A,B**). These events could collectively signify the formation of the Src-like structure. At the final stage, a concerted breakage of the E286-R386 ion pair and reformation of the K271-E286 salt bridge signaled the completion of the conformational transition and stabilization of the active ABL form. These energetic changes may reflect concerted structural motions of the activation loop and αC-helix that could collectively serve as mechanistic wheels of the activation transitions (**Figures 13** **,** **14**).

**Figure 14:**
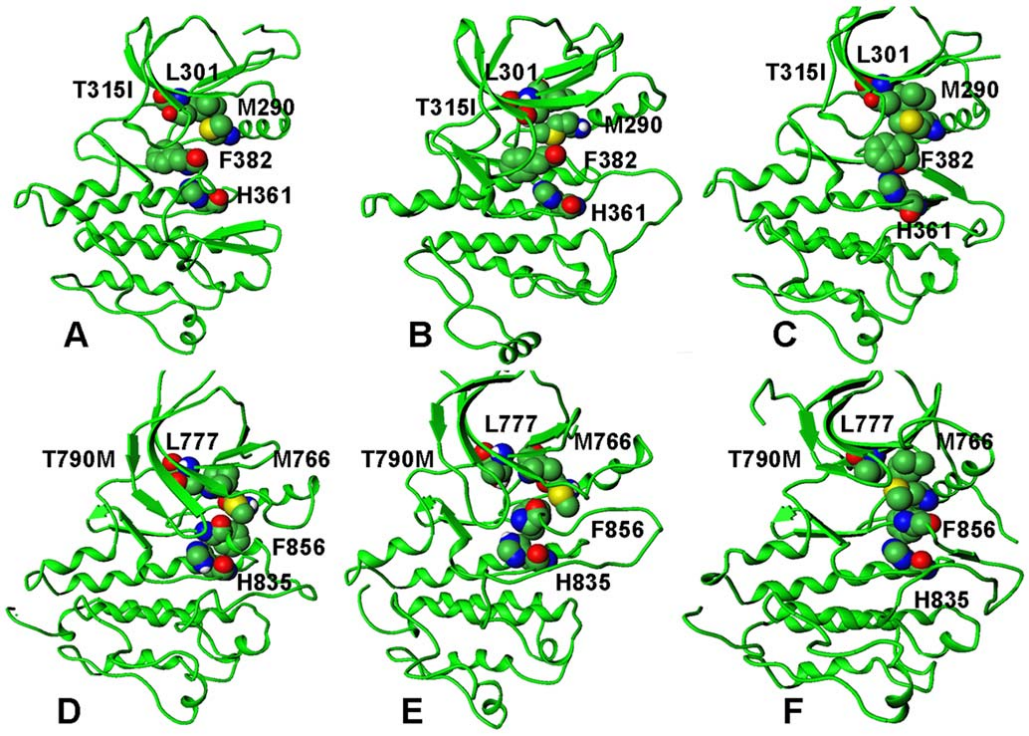
A mechanistic analysis of the TMD activation pathways for the ABL-L387M and EGFR-L858R mutations. A dynamic assembly of the hydrophobic spine during TMD simulations is shown for the ABL-L387M mutant (upper panel) and the EGFR-L858R mutant (lower panel). (A) The initial model of ABL-L387M in the inactive form. (B) The characteristic TMD intermediate of ABL-L387M. (C) The final structure of ABL-L387M in the active form. (D) The initial model of EGFR-L858R in the inactive form. (B) The characteristic TMD intermediate of EGFR-L858R. (C) The final structure of EGFR-L858R in the active form. The assembled hydrophobic spine is present in all TMD intermediates.

### Structural Analysis of Activation Pathways in the ABL and EGFR Kinase Domains

We analyzed mechanistic features of the ABL/EGFR activation pathways that suggested interesting universal aspects of the activation mechanism (**Figure S5**). We detail this analysis by highlighting crucial elements of ABL conformational transitions. Initially, the “upper” part of the activation loop (residues 379–383) remained relatively stable and unperturbed by the activation process (**Figure S5, upper panel**). At the same time the “middle” region of the activation loop (residues 384–391) began to unfold towards a more open conformation. In particular, the kinase residues 384–387 moved closer to the P-loop. As a result, the distance between Arg386 (activation loop) and Glu 255 (P-loop) decreased and then started increasing as the activation loop moved towards the intermediate Src-like inactive conformation. The middle segment (residues 387–391) led the transition of the activation loop towards the Src-like intermediate state. This process was supported by the unfolding of the anti-parallel β-sheet at the lower end of the activation loop (residues 394–400). The activation loop continued its movement towards the Src-like inactive conformation by pushing αC-helix away from the active site to allow itself a sufficient room between αC-helix and the catalytic loop, while the P-loop came in to fill the created gap (**Figure S5**).

Importantly, we found a high degree of similarity between a meta-stable TMD intermediate and the Src-like crystal structure (**Figure S6**). The main structural features of the Src-like ABL conformation were reproduced in the TMD intermediate state. In this conformation, αC-helix was rotated and moved out of the active site (αC-helix-Glu-out position), the DFG motif flipped in the intermediate DFG-in position, the anti-parallel β-sheet from the lower part of the activation loop unfolded and the P-loop moved in towards the active site (**Figure S6**). These structural changes were accompanied by a concerted breakage of the K271-E286 ion pair and formation of the E286-R386 salt bridge. Hence, the molecular mechanism of ABL activation by cancer mutations may favor stabilization of the Src-like structure.

### Mechanistic Catalysts of the EGFR Kinase Domain Activation

The EGFR-L858R mutation is recognized for its frequent occurrence and high oncogenic activity and may arguably have a more drastic effect on the activation mechanism. Indeed, what attracted our attention in the analysis of TMD simulations was a pronounced, “first-order”-like transition in EGFR L858R at the onset of 4 ns. At this point K745-E762 and E762-R858 distances sharply decreased and respective pairs came considerably closer, yet none of these ion pairs was fully formed (**Figure 12D**). Intrigued by this observation, we inspected the representative conformations immediately preceding and following this drastic transition, in order to gain structural insight into the nature of meta-stable intermediates. A considerable conformational change and formation of a meta-stable intermediate was coordinated by repositioning of the αC-helix towards the active-like state. Hence, EGFR-L858R may facilitate the activation process through coordinated assembly of the hydrophobic spine and movements of αC-helix toward the active-like position. While the hydrophobic spine seemed to be established at the transition state conformations, the K745-E762 salt bridge was already broken and the E762-R858 ion pair was not yet formed . During a second coordinated transition to the active kinase form, we detected a concerted formation of the K745-E762 salt bridge and a simultaneous disengagement of the E762-R858 pair (**Figure 12D**).

Hence, consolidation of the hydrophobic spine and formation of the K745-E762 salt bridge are main characteristics of both normal and oncogenic EGFR forms, as was previously suggested. A cooperative “mechanical lock” staples the gatekeeper T790 residue with the hydrophobic spine in the EGFR kinase domain (Met766, Leu777, His835, and Phe856) (**Figure 14**). Hence, consolidation of the hydrophobic spine and formation of the K745-E762 salt bridge are main characteristics of both normal and oncogenic EGFR forms, as was previously suggested [71]. Importantly, our analysis, which is based on sufficiently longer TMD simulations, revealed a different and more subtle mechanistic role of these structural catalysts in the activation process. Indeed, a rapid formation of the hydrophobic spine corresponded to the early first sharp transition and may serve as a key structural “trigger” of the activation process that is accelerated by the mutation. Subsequently, strengthening of the K745-E762 salt bridge that occurred at the late stage of the reaction may function as the structural “consolidator” sealing a complete assembly of the active kinase form. Combining thermodynamic and mechanistic insights, we could suggest that a pronounced effect of EGFR-L858R on the activation process may partly explain the unusually high oncogenic activity and frequent occurrence of L858R in lung cancer and a high oncogenic activity of the T790M/L858R tandem [50]. These results corroborated with our previous insights, suggesting that cancer mutations may have a similar mechanism of activation, yet mutations with the higher oncogenic activity may have a greater differential impact on thermodynamic stability of the active and inactive states. Molecular mechanisms of activation by cancer mutations could mimic the activation process of the normal kinase, while exploiting conserved structural catalysts to accelerate a conformational transition and the enhanced stabilization of the active kinase form.

A rigorous structural and energetic analysis of the transition state ensemble (TSE) for the activation reaction implies the determination of the “true” reaction coordinate and computations of the potential of mean force (PMF) along the respective activation pathways [67],[68]. A comparative analysis of the TSE for studied systems would require considerable computational resources and extends beyond the scope of this study. Nevertheless, by combining thermodynamic and mechanistic insights obtained in this study, we could offer a plausible qualitative rationale of the kinetic factors contributing to the kinase activation. In particular, we could speculate that the observed structural assembly of the hydrophobic spine may control energetic stabilization of the TSE during the activation reaction in both normal and oncogenic kinase forms. This may effectively lower the activation barrier for bulkier gatekeeper mutants that could strengthen their interactions in the TSE with the hydrophobic regulatory spine. Concurrently, the differential thermodynamic effect of mutations may destabilize the inactive kinase form and also contribute to lowering of the activation energy barrier. It is likely that these factors could collectively enhance kinase activation by cancer mutations.

### Molecular Docking and Differential Sensitivity of EGFR Inhibitors

Understanding of molecular signatures of activation mechanisms in the normal and oncogenic states may aid in the correlation of mutational effects with the inhibitor binding. The available crystal structures and experimental dissociation constants of the EGFR complexes with cancer drugs provided a basis for computational modeling using molecular docking and binding free energy simulations. The primary objective of this analysis was to offer a molecular rationale to the observed differential sensitivity by which EGFR-T790M and EGFR-L858R mutants confer to Lapatinib [23], Geftinib, and AEE788 inhibitors [51]–[53] (**Figure S7**). The predicted binding mode of Lapatinib bound to the co-crystal inactive structure and computed binding affinity were in a good agreement with the crystal structure (**Figure S8A**) and experimental dissociation constant (**Table S1**). In accordance with the thermodynamic data, Lapatinib was highly selective towards the inactive form of the WT EGFR, since the predicted binding poses with the active EGFR structures resulted in structural divergence and a concomitant dramatic loss in the binding free energies (**Table S1**). Docking of Geftinib with the EGFR crystal structures predicted binding modes of the inhibitors within RMSD = 1.5 Å from the respective crystal structures (**Figures S8 B**). In agreement with the crystallographic data, docking of AE788 with the spectrum of EGFR structures predicted virtually identical binding modes of the inhibitor (**Figure S8 C**). The predicted binding mode of Geftinib in the EGFR-T790M crystal structure suggested a molecular evidence for the greater potency of the inhibitor over EGFR-WT. Indeed, the enhanced interactions between the aniline ring of Geftinib and the M790 residue side-chain may contribute to the greater potency of this inhibitor towards the T790M EGFR mutant (**Figure S8 D, E**). Indeed, the more favorable halogen-sulfur interactions formed between Geftinib and M790 in the crystal structure resulted in the greater potency of this inhibitor, as compared to AEE788, which was consistent with the experimental data (**Figures S8 F, Table S1**). Structural similarity of the inhibitor complexes with the active forms of EGFR produced respectively similar binding free energies, as manifested in the binding profile of Geftinib (**Table S1**). Hence, the enhanced binding affinity of Geftinib to the L858R and T790M mutants could be attributed to weaker binding with the inactive EGFR-WT, rather than to the differences in the inhibitor binding with the active form of the kinases. These results suggested that structural preponderance of the active form for the studied EGFR mutants may be an important driving factor of their enhanced drug binding.

### Conclusions and Implications

This study reconciled current structural and functional data with the insights from computational approaches, pointing to general mechanistic aspects of activating transitions in protein kinases. In particular, the analysis of activation pathways in the ABL and EGFR kinase domains has revealed common features that qualitatively agree with the earlier studies of activating conformational transitions in the Src kinase [64]–[67] and CDK5 kinase [68]. Interestingly, the formation of Src-like intermediate structure that may facilitate conformational transitions between inactive and active kinase forms may be a common feature of activation mechanism. Indeed, a very similar low-energy intermediate conformation was also observed in simulations of CDK5 kinase [68]. The observed similarity in the nature of the conformational pathways and structural features of the intermediate conformations shared in a number of kinases may suggest functional significance of the proposed activation mechanism in kinase regulation. The emerging understanding of molecular signatures, associated with cancer causing mutations in protein kinases may ultimately help in tracing the effect of genetic variations responsible for the molecular pathologic ‘lesions’ associated with disease susceptibility.

Although the value of protein structural information in the design of cancer therapeutics, for which specific mutations are likely to be the cause, has been recognized [91],[92], structure-based drug design has rarely exploited such information. Though understanding of the precise role of genetic alterations in tumorigenesis is challenging, exploiting personal genomic data may provide a basis for discovery of cancer drugs targeting a spectrum of mutational changes that occur in cancer [93],[94]. Characterization of the conformational landscape for the protein kinases and cancer mutants can provide access to unique conformations that are rarely exploited in biochemical and structural experiments typically biased towards the active state of the enzyme. This could facilitate design of kinase inhibitors selectively targeting kinase activation pathways as well as engineering and optimizing the clinical effects of existing drugs. Hence, future computational modeling studies can further inform and facilitate experiments exploring the molecular pathology of tumorigenesis and implications in rationale drug design of specific cancer therapies.

## Materials and Methods

### Structure Preparation

In simulations of the ABL and EGFR kinase domains, we used the following crystal structures from the Protein Data Bank (PDB): pdb entry 1IEP (inactive ABL structure), pdb entry 2G1T (Src-like inactive ABL structure), pdb entry 1M52 (active ABL structure), pdb entry 1XKK (Src/Cdk-like inactive EGFR structure), pdb entry 2GS7 (Src/Cdk-like inactive EGFR structure) and pdb entry 2J6M (active EGFR structure). For simulations with the ABL complexes, we used the crystal structure of the ABL-SH2-SH3 complex in the inactive form (pdb entry 2FO0) and active form (pdb entry 1OPL). For simulations with the EGFR dimers, we utilized the crystal structure of the EGFR in the inactive state (pdb entry 2GS7). All crystallographic water molecules, bound inhibitors, and other heteroatoms were removed. The residue range 702–984 was used for the EGFR kinase domain and 225–498 for the ABL kinase domain. The structure of 1XKK is unresolved for residues 749–754 (activation loop) and residues 867–876 (αC-helix), thus the unresolved parts were modeled using the program MODELLER which is an automated approach to comparative protein structure modeling by satisfaction of spatial restraints [95],[96]. Similarly the active form of ABL (pdb entry 1M52) does not have residues 225–231, so they were also modeled using the same strategy. All mutations were introduced into the respective crystal structures of ABL and EGFR using MODELLER. Local minimization was done to relax the protein environment surrounding the mutant residue.

### Homology Modeling

Homology modeling of the ABL and EGFR mutants was done using MODELLER 95,96 with a subsequent refinement of side-chains by the SCRWL3 program [97]. Initial models were built based on the crystal structures of ABL-WT and EGFR-WT in inactive, Src-like inactive and active forms. The mutated structures were initially refined using 5000 steps of minimization to ensure sufficient relaxation of the local environment near mutational site. Initial models were built in MODELLER with a flexible sphere of 5 Å around mutated residue. We gradually increased the radius of this sphere in 5 Å steps until the radius reached the 25 Å values (i.e. all residues falling within this range were treated as flexible). A protocol involving a conjugate gradient (CG) minimization, followed by simulated annealing refinement was repeated 20 times to generate 100 initial models for each cancer mutant in this study. In the optimization stage, we initially used CG minimization to remove unfavorable contacts and to optimize geometry. MD simulations were then run at increasing temperature values from 150 K to 1500 K, followed by simulated annealing and sampling at temperatures of 1500 K, 1000 K, 800 K, 600 K, 500 K, 400 K, 320 K, and 300 K respectively. The models were generated using 20 iterations of this protocol and the predicted structural model was chosen out of the 100 models as scored by the MODELLER default scoring function. The final models were then refined in 2 ns MD simulations using NAMD 2.6 [98] with the CHARMM27 force field [99],[100]and the explicit TIP3P water model as implemented in NAMD 2.6 [101].

### MD Simulations

MD simulations of the ABL kinase domain were independently launched for ABL-WT, ABL-T315I and ABL-L387M from the inactive form (pdb entry 1IEP), Src-like structure (pdb entry 2G1T) and active state (pdb entry 1M52). Similarly, MD simulations of the EGFR kinase domain were initiated for EGFR-WT, EGFR-T790M, EGFR-L858R from the Src/Cdk-like inactive structures (pdb entries 1XKK, 2GS7), and active conformational form (pdb entry 2G6M). We performed a total of 15 MD simulations of the ABL and EGFR kinase domains (each of 10 ns duration) and a total of 4 MD simulations of the ABL and EGFR complexes (each of 20 ns duration). MD simulations were carried out using NAMD 2.6 with the CHARMM27 force field [102],[103] and the explicit TIP3P water model widely used in MD simulations [101]. The details of structure preparation and solvent models are given in **Tables S2** and **S3**. The VMD program was used for the preparation and analysis of simulations [102].

The psfgen utility in VMD was employed to generate a protein structure file. The residue Tyr-393 from the ABL kinase domain (all structures) and Tyr-869 in the EGFR kinase domain (all structures) were phosphorylated (−2 charge state) using VMD. Thereafter the structures were solvated in a box of water with the buffering distance of 15 Å; assuming normal charge states of ionizable groups corresponding to pH 7, sodium (Na^+^) and chloride (Cl^−^) counter-ions at physiological concentration of 0.15 mol/L were added to achieve charge neutrality and to mimic biological environment more closely. All Na^+^ and Cl^−^ ions were placed at least 8 Å away from any protein atoms and from each other. The system was subjected to initial minimization for 10,000 steps keeping protein atoms fixed (minimization of water molecules) followed by 10,000 steps of minimization keeping only protein backbone fixed to allow protein side chains to relax. This was followed by final 10,000 steps of minimization without any constraints on the system.

Equilibration was done in steps by gradually increasing the system temperature in steps of 20 K starting from 10 K until 310 K. At each step 10,000 steps equilibration was run while applying a harmonic restraining force of 10 Kcalmol^−1^ Å^−2^ to all backbone C_α_ atoms. Thereafter the system was equilibrated for 150,000 steps at 310 K (NVT) and then for further 150,000 steps at 310 K using Langevin piston (NPT) to maintain the pressure. Finally the restrains were removed and the system was equilibrated for 500,000 steps to prepare the system for simulation.

An NPT simulation was run on the equilibrated structure keeping the temperature at 310 K and pressure at 1 bar using Langevin piston coupling algorithm. The integration time step of the simulations was set to 2.0 fs; the SHAKE algorithm was used to constrain the lengths of all chemical bonds involving hydrogen atoms at their equilibrium values; and the water geometry was restrained rigid by using the SETTLE algorithm. Nonbonded van der Waals interactions were treated by using a switching function at 10A and reaching zero at a distance of 12 A. The particle-mesh Ewald algorithm (PME) as implied in NAMD was used to handle long range electrostatic forces [103]. The entire procedure was applied to all structures used in this study.

### Protein Stability Analysis

Protein stability calculations were done using the molecular mechanics (MM) AMBER force field [104] and the solvation energy term based on continuum generalized Born and solvent accessible surface area (GB/SA) solvation model [105]–[108]. In MM-GBSA calculations, we employed AMBER99 force field. The choice of AMBER99 was made because we used phosphorylated tyrosine residues in MD simulations (Tyr-393 in ABL and Tyr-869 in EGFR) for which available parameters were derived in the AMBER94/99 force field. The parameters for phosphorylated tyrosines were taken from [108].

The total free energy was given as
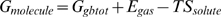

*G_gbtot_* is the solvation free energy; *E_gas_* is the molecular mechanical energy of the molecule summing up the electrostatic *Eele* interactions, the van der Waals contributions *E_vdw_* and the internal strain energy *E_int_*. *TS_solute_* is the sum of *TS_trans_*, *TS_rot_*, and *TS_vib_* which are the translational, rotational and vibrational entropy contributions. The non-polar contribution to the solvation free energy *G_gbnp_* was determined using the Linear Combinations of Pairwise Overlaps (LCPO) method where the hydrophobic contribution to the solvation free energy is determined by the Solvent Accessible Surface Area (SASA) dependent term. The total solvation free energy *G_gbtot_* is the sum of nonpolar *G_gbnp_* and polar *G_gbele_* contributions. The electrostatic contribution to the solvation free energy Ggbele involves using the GB equation to estimate the electrostatic contributions to the solvation free energy. We also carried out normal mode calculations to determine the entropy contribution to the protein stability changes. Normal mode analysis involves calculation and diagonalization of a mass-weighted second derivative matrix. The entropic contribution was calculated within the AMBER module NMODE, where the vibrational entropy contribution TS_vib_ was evaluated from classical statistical mechanics formula. A dielectric constant of 4r_ij_, where r_ij_ is the distance between atoms i and j of the molecule, is used in the normal mode calculations.

Assuming a two state model for calculation of the free energy stabilities, we would need to evaluate the difference in energy between the folded and unfolded WT kinase and compare to the respective difference in energy between the folded and unfolded mutant kinase. It is assumed that, in the unfolded state, individual residues may not interact, and hence the contributions of residues other than the one under mutation are the same in the WT and mutant proteins. Hence, protein stability computations for the WT and mutant kinases could refer to somewhat different unfolded reference states, which are associated with the free energies of individual WT and mutated residues in solution. According to our model, we assumed that this difference could be small compared to the respective differences between free energies of folded WT and mutant kinase structures.

Consequently, we have evaluated protein stability changes induced by mutations in structurally different conformational forms of ABL and EGFR. A practical implementation of this approximation involved evaluation of the free energies for 1,000 snapshots selected at 10 ps interval along MD trajectories for the WT and respective mutant. The total free energy values were obtained by averaging calculated contributions over 1,000 simulation snapshots. The protein stability changes were then approximated based on the free energy difference between the WT and mutant kinase. Free energy evaluations could be performed using either separate trajectories of the WT and mutant kinases or a single trajectory of the WT protein, followed by introducing mutation and local refinement of the individual snapshots from the WT trajectory [109]. We have used a more rigorous, yet more computationally intensive separate trajectory protocol based on independent MD simulations of the WT and mutant kinases.

### TMD Simulations

In TMD simulations, a chosen subset of atoms is steered towards a target structure, while the other atoms may also move in response to this structural change. Hence, this approach could simulate large scale conformational transition in biomolecules which are otherwise difficult or impossible to realize using conventional MD simulations. The RMSD between the current structure and the target structure was used to calculate an additional steering force in TMD simulations, which was applied on every atom from the chosen molecular subset, and is calculated as follows:
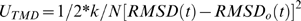
RMSD(t) represents the relative distance for a chosen molecular subset between the current structure at the simulation time t and the reference target structure. RMSD_0_ (t) is the target RMSD value at simulation time t. The technical details of TMD simulations setup were similar to equilibrium MD simulations. The important distinction is presence of the RMSD restraint with a force constant of 2 Kcalmol^−1^ Å^−2^ that was applied to all heavy atoms in the activation loop. TMD simulations were run for 10 ns with a time step of 2 fs. The conformational transition from the initial to the target structure was determined by decreasing the value of RMSD_o_ (t) as a function of the simulation time. The value of RMSD_o_ (t) was linearly decreased to 0 Å during the first 2 ns of TMD, and then kept at zero during the subsequent 10 ns of equilibration. Upon reaching the target structure, an additional 2 ns allowed to fully relax into the final target structure.

The complete activation loop (residues 379–401 in ABL and residues 853–878 in EGFR) was a subject of a steering force which was applied to every atom of this protein region to simulate conformational transitions between inactive and active kinase forms. TMD simulations were carried out for ABL-WT, ABL-T315I and ABL-L387M to model conformational transitions from the Imatinib-bound, inactive state of ABL (pdb entry 1IEP) to the target active ABL conformation (pdb entry 1M52 for ABL-WT and ABL-L387M; pdb entry 2Z60 for ABL-T315I). Similarly, TMD simulations were carried out for EGFR-WT, EGFR-T790M and EGFR-L858R to model conformational transitions between the Src/Cdk-like inactive form of EGFR (pdb entry 1XKK) and the target active EGFR form (pdb entry 2J6M for EGFR-WT; pdb entry 2JIU for EGFR-T790M; and pdb entry 2ITT for EGFR-L858R). The details of structure preparation for TMD simulations of the ABL and EGFR kinase domains are given in **Table S2**.

### Molecular Docking and Binding Free Energy Calculations

Molecular docking simulations of EGFR inhibitors were performed using replica-exchange Monte Carlo simulations with the ensembles of multiple protein kinase conformations. The protein conformational ensembles for the EGFR-WT, EGFR-T790M and EGFR-L858R were obtained by extracting a total of 1,000 snapshots from MD simulations with different conformational forms of the EGFR kinase domain. The details of the docking protocol were documented in details in our previous studies [110],[111].

In brief, we employed the AMBER force field combined with an implicit solvation model. The dispersion-repulsion and electrostatic terms were modified to include a soft core component that was originally developed in free energy simulations to remove the singularity in the potentials and improve numerical stability of the simulations [112]. Replica-exchange simulations used 1,000 replicas of the system (corresponding to 1,000 different snapshots) attributed respectively to 1,000 different temperature levels that were uniformly distributed in the range between 5300 K and 300 K. Monte Carlo moves were performed simultaneously and independently for each replica at the corresponding temperature level. A process of swapping configurations was repeated 100 times after each simulation cycle for all replicas. The inhibitor conformations and orientations were sampled in a parallelepiped that encompasses the crystal structures of the inhibitor complexes with a 10.0 Å cushion added to every side of the box surrounding the binding interface. The protein kinase conformation was held fixed in the minimized conformation, while the rigid body degrees of freedom and the inhibitor rotatable angles were treated as independent variables during ligand docking.

The binding free energy of the protein kinase complexes with inhibitors was calculated using the following expression:
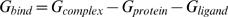
Binding free energy evaluations were performed by using the distribution of 1,000 low-energy complexes obtained from replica-exchange Monte Carlo docking simulations at T = 300 K. The structures for the uncomplexed kinase and the inhibitor were generated by separating the protein and inhibitor coordinates, followed by an additional minimization of the unbound protein and inhibitor.

## Supporting Information

Figure S1 Conformational Landscape of the ABL Kinase Domain. The crystal structures of ABL represent the following conformational forms: the Imatinib-bound, inactive structure (pdb entry 1IEP) (A), the Src-like inactive structure (pdb entry 2G1T) (B), and the active structure (pdb entry 1M52) (C). A conserved K271-E286 salt bridge is characteristically present in the Imatinib-bound inactive structure (D). This salt bridge is broken and replaced by the E286-R386 ion pair in the Scr-like inactive structure (E). The K271-E286 ion pair is restored in the active structure (F). The activation loop in these structures is highlighted in blue and the activating mutations are denoted by red balls.
(1.97 MB TIF)

Figure S2 Conformational Landscape of the EGFR Kinase Domain. The crystal structures of EGFR represent the following conformational forms: the Lapatinib-bound, inactive structure (pdb entry 1XKK) (A), the Src/Cdk-like inactive structure (pdb entry 2GS7) (B), and the active structure (pdb entry 2J6M) (C). A conserved K745-E262 salt bridge is broken in the Src/Cdk-like inactive structures (D,E). This salt bridge is present in the active structure (F). The activation loop in these structures is highlighted in blue and the activating mutations are denoted by red balls.
(2.07 MB TIF)

Figure S3 A Close-up of the Structural Cluster Formed by the Gatekeeper T315I Mutant and the DFG Motif. (A) Superposition of the predicted structural cluster between ABL-T315I and DFG (in blue) with the crystallographic conformation (pdb entry 2Z60, in green). (B) Superposition of the predicted structural cluster between EGFR-T790M and DFG (in blue) with the crystallographic conformation (pdb entry 2JIT, in green) (C) Similarity in the predicted structure and packing interactions between the DFG conformation and the gatekeeper residues T315I in ABL (dark blue) and T790M in EGFR (light blue). Note that the predicted EGFR-DFG conformation is similar to the one observed in the second molecule of the EGFR-T790M crystal structure, which fully overlaps with the predicted model.
(0.44 MB TIF)

Figure S4 MD Simulations of the symmetric EGFR dimer. Upper Panel: Color-coded mapping of the averaged protein flexibility profiles (RMSF values) from MD simulations of the symmetric EGFR dimer (pdb entry 2GS7). The mapping is presented for EGFR-WT (A) and EGFR-T790M (B). The color-coded sliding scheme is the same as was adopted for Figure 3. Lower Panel: The RMSD fluctuations of Cα atoms (C) and the RMSF values of Cα atoms (D) from MD simulations. MD simulations of EGFR-WT (in blue), and EGFR-T790M (in red) were performed using the inactive EGFR dimer (pdb entry 2GS7).
(0.65 MB TIF)

Figure S5 Overview of the TMD Activation Pathways in ABL Kinase. A mechanistic view of the activation process using representative snapshots along the activation pathway is shown on the upper panel for ABL-WT (A), ABL-T315 (B), ABL-L387M (C), and on the lower panel for EGFR-WT (D), EGFR-T790M (E), and EGFR-L858R (E). The kinase domain is shown in grey. The activation loop conformations along activation pathways are highlighted. Structural changes along the pathway are reflected in collective motions of the activation loop and αC-helix, serving as mechanistic “wheels” of the reaction.
(2.05 MB TIF)

Figure S6 Structural similarity between TMD intermediate and the Src-like crystal structure of ABL. A high similarity is found between a meta-stable intermediate formed in TMD simulations of ABL and the Src-like ABL crystal structure. (A) An overlay between the Imatinib-bound crystal structure of inactive ABL (in blue) and the Src-like inactive crystal structure of ABL (in red). Note that the β-sheet formed in the inactive form is broken in the Src-lime structure. (B) An overlay between the Src-like inactive crystal structure of ABL (in red) and a meta-stable TMD intermediate (in yellow). Note that the important structural features of the Src-like inactive ABL conformation were reproduced in the TMD intermediate state.
(1.25 MB TIF)

Figure S7 Chemical Structures of Lapatnib, Geftinib and AE788 Inhibitors.
(0.26 MB TIF)

Figure S8 The Predicted Binding Modes of Lapatinib, Geftinib and AE788. The crystallographic conformations of Lapatinib (A), Geftinib (B), and AE788 (C) are shown in default colors (bold, stick). The crystal structure of the inhibitor is superimposed with the predicted binding poses docked into the inactive EGFR-WT (yellow stick), active EGFR-WT (blue stick), EGFR-T790M (light blue stick) and EGFR-L858R (dark green stick). (D) A close-up of interactions between Geftinib and T790 in the WT EGFR crystal structure. (E) A close-up of interactions between Geftinib and T790 in the WT EGFR crystal structure. (F) A close-up of binding modes and interactions formed by Geftinib (in s blue) and AE788 (in default colors) in the hydrophobic pocket of the T790M EGFR mutant. The phenethylamine substituent of AE788 and chlorine-substituted aniline in Geftinib occupy a similar space in the hydrophobic pocket of the T790M EGFR mutant.
(1.00 MB TIF)

Table S1 A comparison of the experimental and computed binding affinities for the EGFR inhibitors.
(0.03 MB DOC)

Table S2 Structure Preparation Details for MD and TMD Simulations of the ABL/EGFR Kinase Domains.
(0.03 MB DOC)

Table S3 Structure Preparation Details for MD Simulations of the ABL and EGFR Complexes.
(0.03 MB DOC)
