# Supplementary figures and images for: Hierarchical Modeling of Activation Mechanisms in the ABL and EGFR Kinase Domains: Thermodynamic and Mechanistic Catalysts of Kinase Activation by Cancer Mutations
Source: PLoS Comput Biol. 2009 Aug 28;5(8):e1000487. doi: 10.1371/journal.pcbi.1000487 (PMC2722018; doi:10.1371/journal.pcbi.1000487)

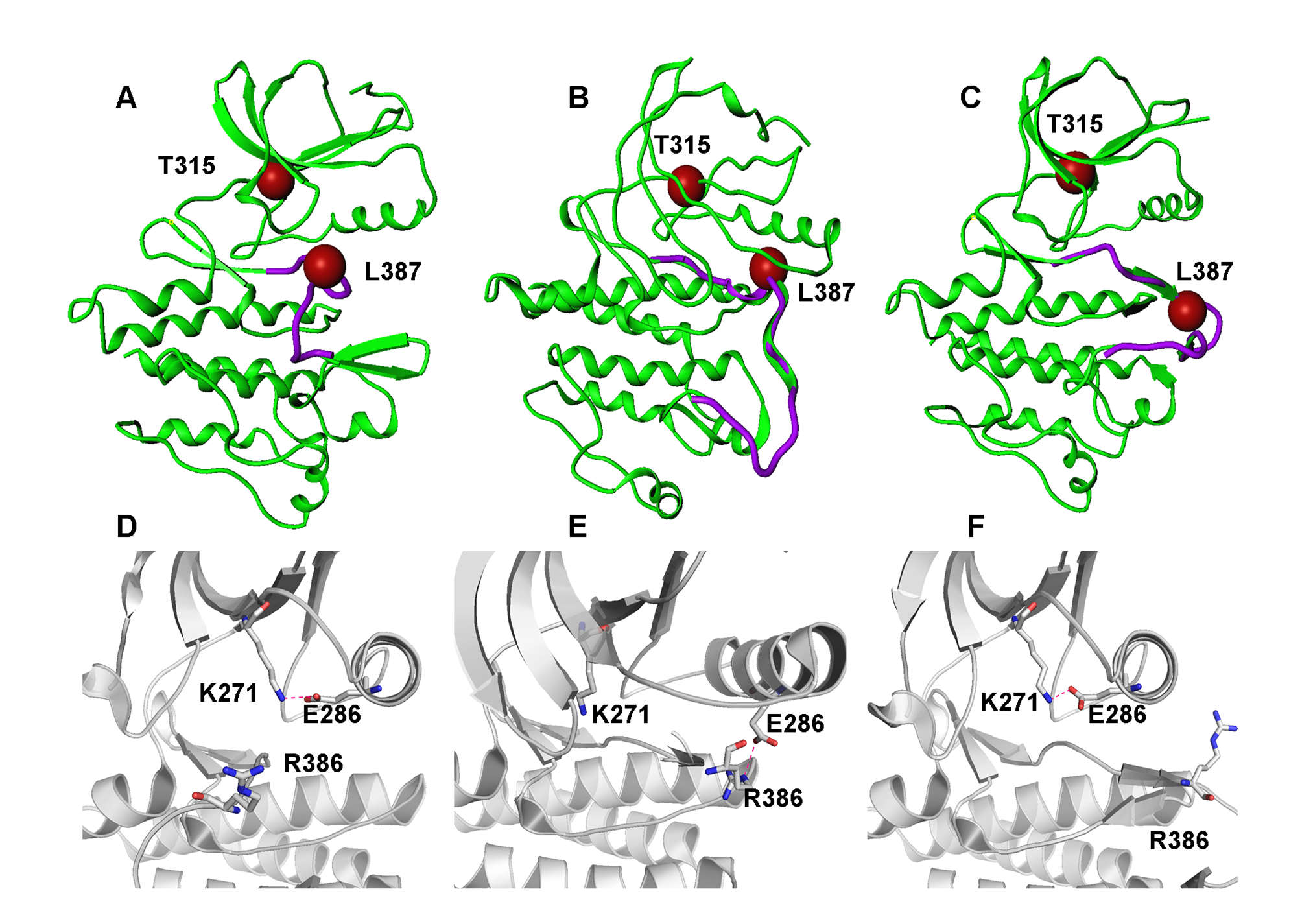

Supplement: Figure S1 — Conformational Landscape of the ABL Kinase Domain. The crystal structures of ABL represent the following conformational forms: the Imatinib-bound, inactive structure (pdb entry 1IEP) (A), the Src-like inactive structure (pdb entry 2G1T) (B), and the active structure (pdb entry 1M52) (C). A conserved K271-E286 salt bridge is characteristically present in the Imatinib-bound inactive structure (D). This salt bridge is broken and replaced by the E286-R386 ion pair in the Scr-like inactive structure (E). The K271-E286 ion pair is restored in the active structure (F). The activation loop in these structures is highlighted in blue and the activating mutations are denoted by red balls. (1.97 MB TIF) [file pcbi.1000487.s001.tif]

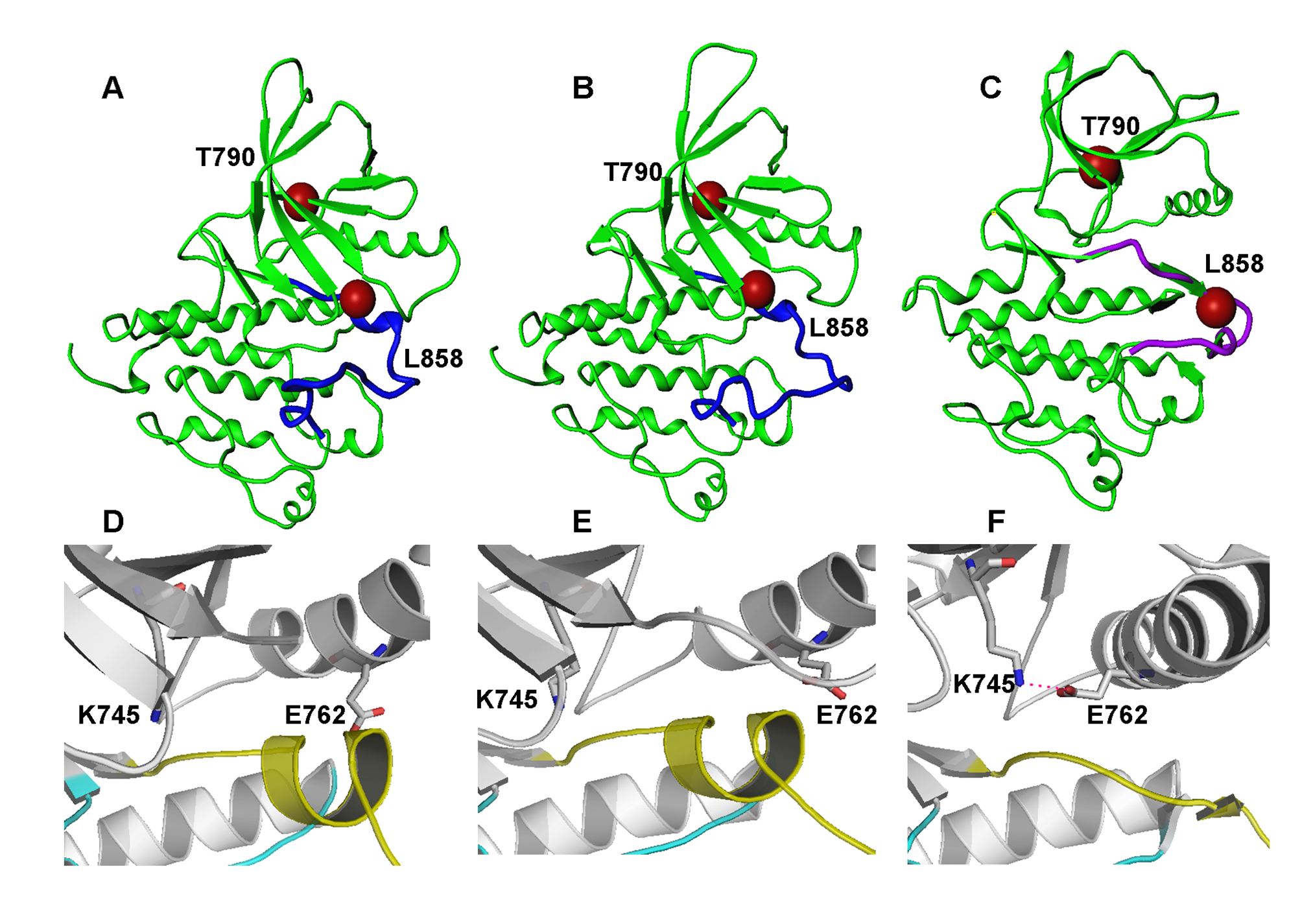

Supplement: Figure S2 — Conformational Landscape of the EGFR Kinase Domain. The crystal structures of EGFR represent the following conformational forms: the Lapatinib-bound, inactive structure (pdb entry 1XKK) (A), the Src/Cdk-like inactive structure (pdb entry 2GS7) (B), and the active structure (pdb entry 2J6M) (C). A conserved K745-E262 salt bridge is broken in the Src/Cdk-like inactive structures (D,E). This salt bridge is present in the active structure (F). The activation loop in these structures is highlighted in blue and the activating mutations are denoted by red balls. (2.07 MB TIF) [file pcbi.1000487.s002.tif]

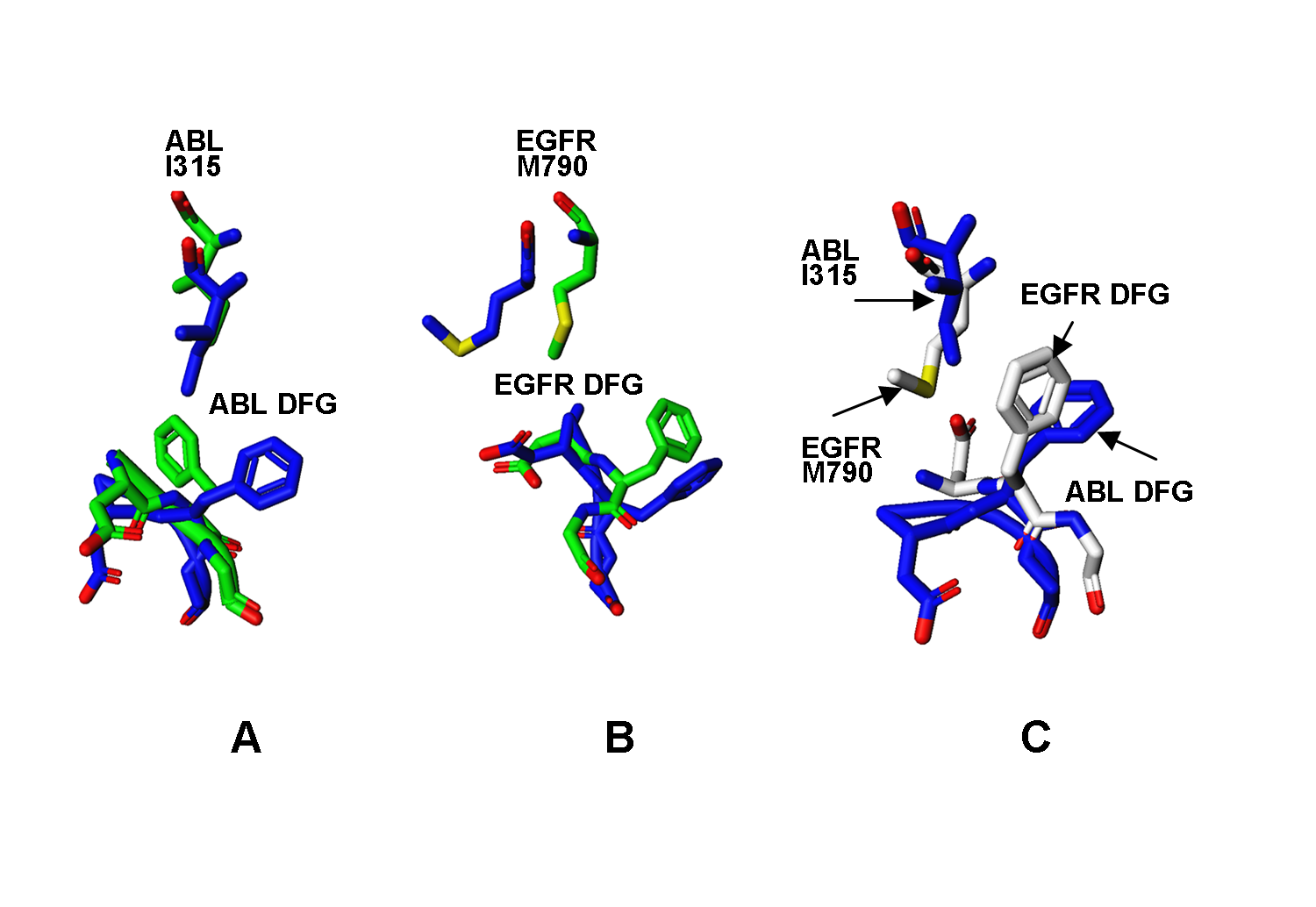

Supplement: Figure S3 — A Close-up of the Structural Cluster Formed by the Gatekeeper T315I Mutant and the DFG Motif. (A) Superposition of the predicted structural cluster between ABL-T315I and DFG (in blue) with the crystallographic conformation (pdb entry 2Z60, in green). (B) Superposition of the predicted structural cluster between EGFR-T790M and DFG (in blue) with the crystallographic conformation (pdb entry 2JIT, in green) (C) Similarity in the predicted structure and packing interactions between the DFG conformation and the gatekeeper residues T315I in ABL (dark blue) and T790M in EGFR (light blue). Note that the predicted EGFR-DFG conformation is similar to the one observed in the second molecule of the EGFR-T790M crystal structure, which fully overlaps with the predicted model. (0.44 MB TIF) [file pcbi.1000487.s003.tif]

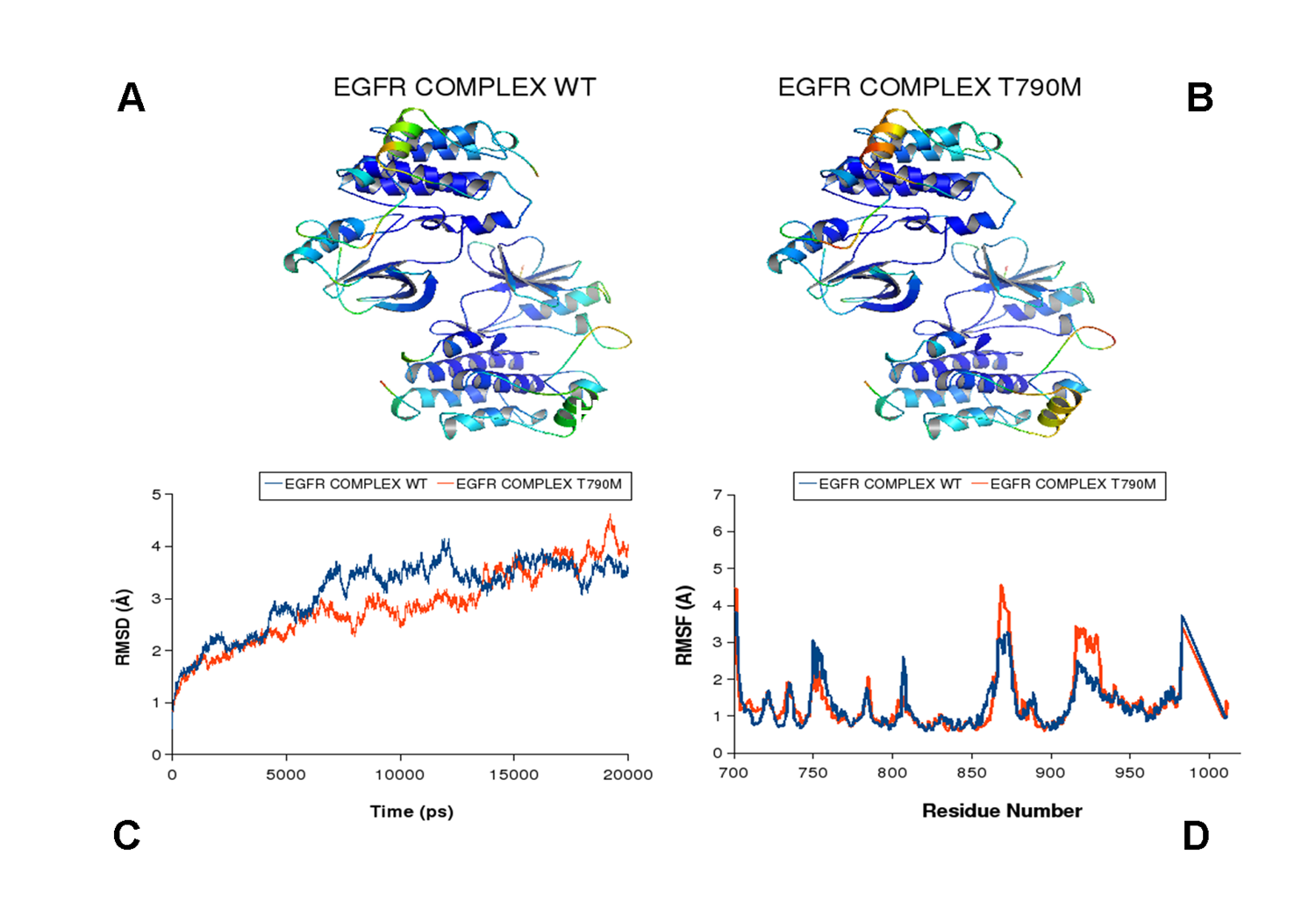

Supplement: Figure S4 — MD Simulations of the symmetric EGFR dimer. Upper Panel: Color-coded mapping of the averaged protein flexibility profiles (RMSF values) from MD simulations of the symmetric EGFR dimer (pdb entry 2GS7). The mapping is presented for EGFR-WT (A) and EGFR-T790M (B). The color-coded sliding scheme is the same as was adopted for Figure 3. Lower Panel: The RMSD fluctuations of Cα atoms (C) and the RMSF values of Cα atoms (D) from MD simulations. MD simulations of EGFR-WT (in blue), and EGFR-T790M (in red) were performed using the inactive EGFR dimer (pdb entry 2GS7). (0.65 MB TIF) [file pcbi.1000487.s004.tif]

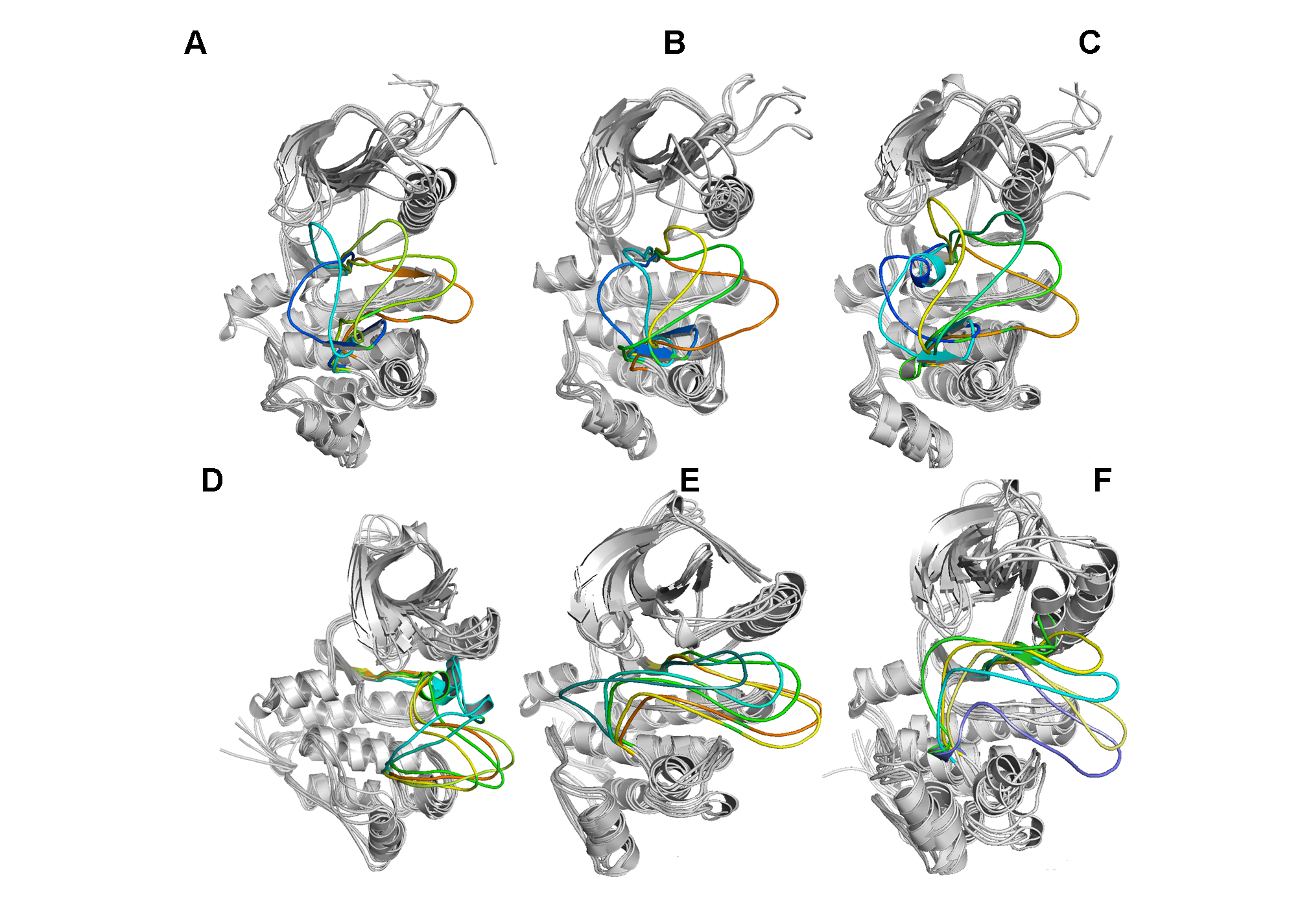

Supplement: Figure S5 — Overview of the TMD Activation Pathways in ABL Kinase. A mechanistic view of the activation process using representative snapshots along the activation pathway is shown on the upper panel for ABL-WT (A), ABL-T315 (B), ABL-L387M (C), and on the lower panel for EGFR-WT (D), EGFR-T790M (E), and EGFR-L858R (E). The kinase domain is shown in grey. The activation loop conformations along activation pathways are highlighted. Structural changes along the pathway are reflected in collective motions of the activation loop and αC-helix, serving as mechanistic “wheels” of the reaction. (2.05 MB TIF) [file pcbi.1000487.s005.tif]

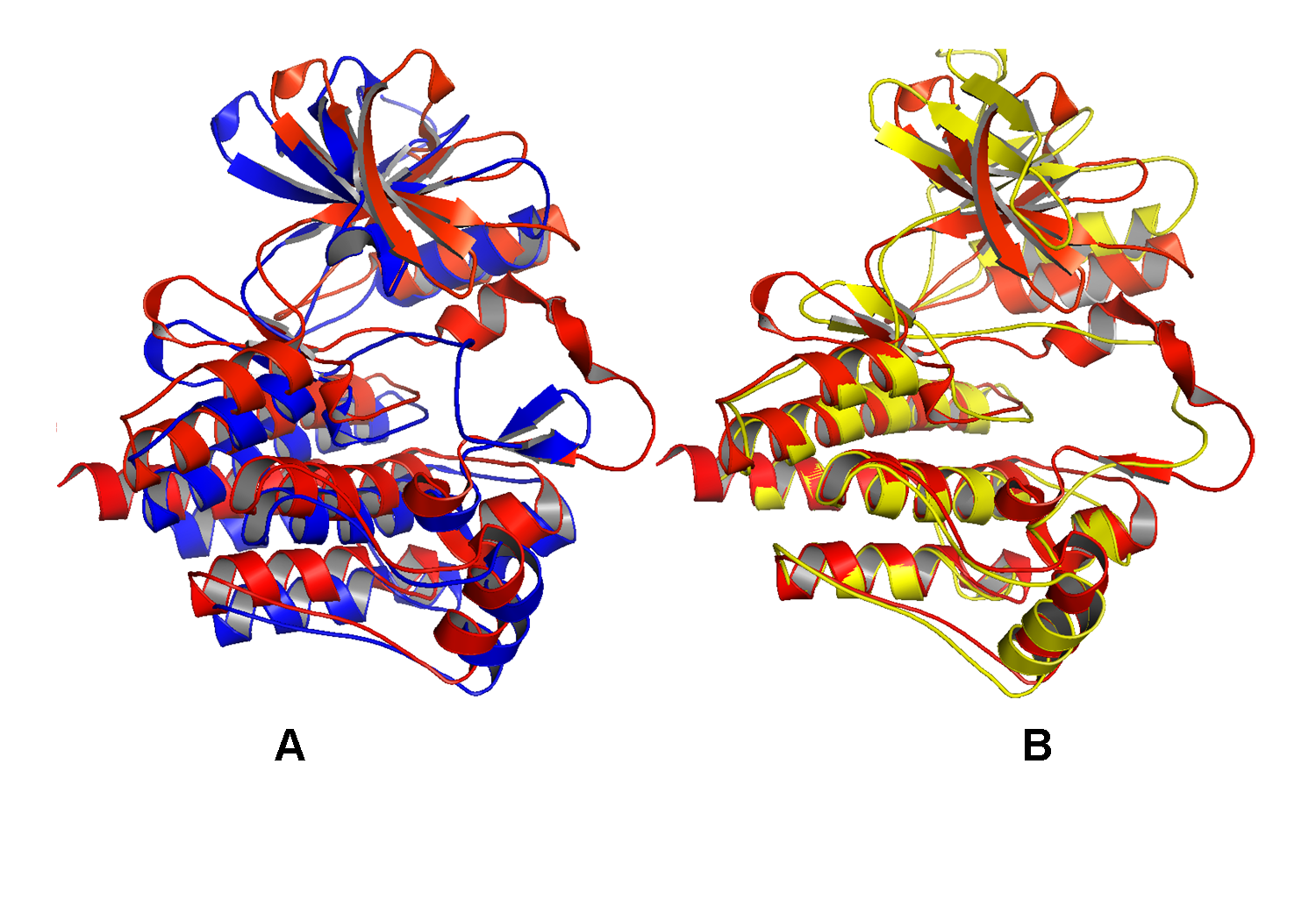

Supplement: Figure S6 — Structural similarity between TMD intermediate and the Src-like crystal structure of ABL. A high similarity is found between a meta-stable intermediate formed in TMD simulations of ABL and the Src-like ABL crystal structure. (A) An overlay between the Imatinib-bound crystal structure of inactive ABL (in blue) and the Src-like inactive crystal structure of ABL (in red). Note that the β-sheet formed in the inactive form is broken in the Src-lime structure. (B) An overlay between the Src-like inactive crystal structure of ABL (in red) and a meta-stable TMD intermediate (in yellow). Note that the important structural features of the Src-like inactive ABL conformation were reproduced in the TMD intermediate state. (1.25 MB TIF) [file pcbi.1000487.s006.tif]

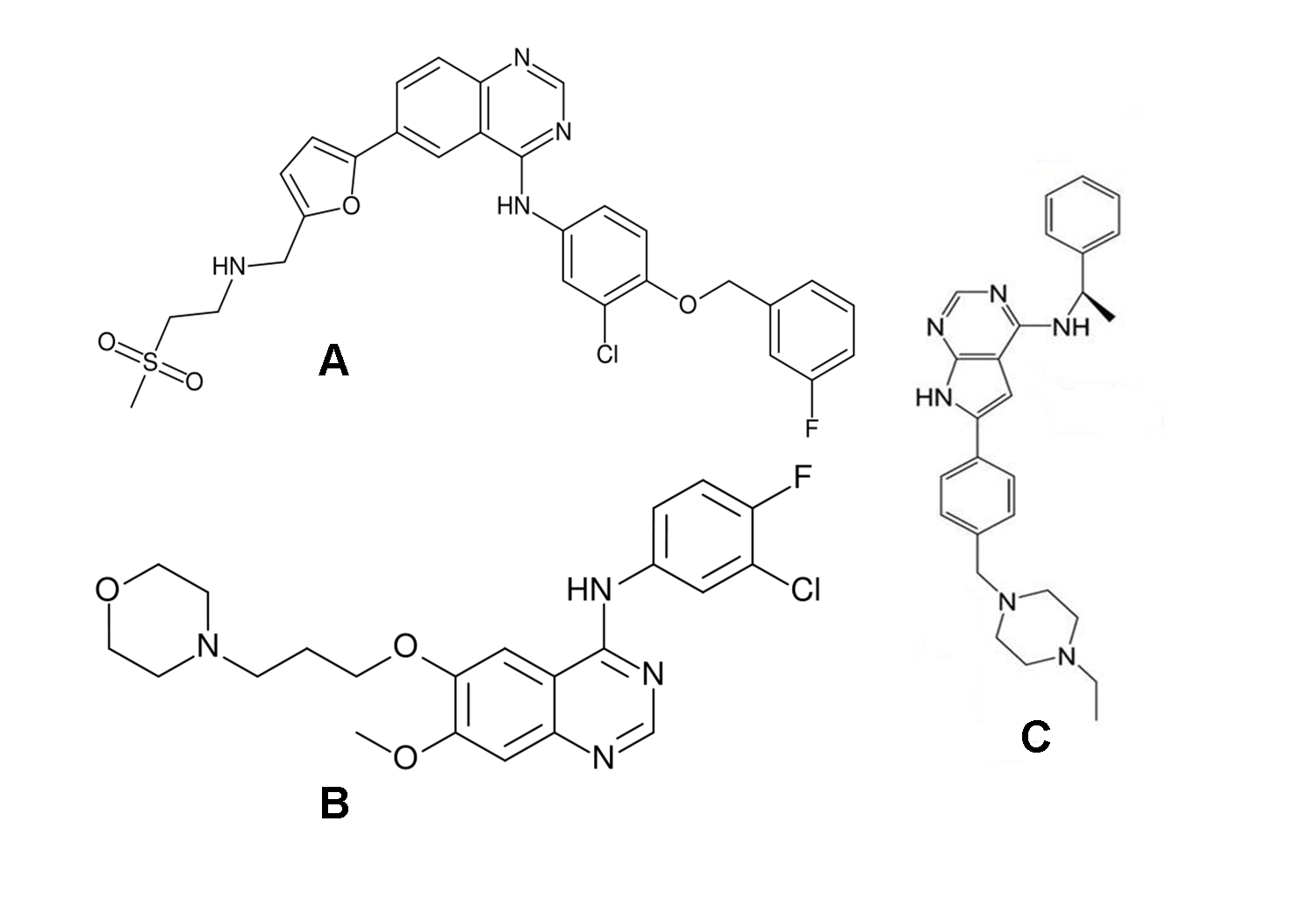

Supplement: Figure S7 — Chemical Structures of Lapatnib, Geftinib and AE788 Inhibitors. (0.26 MB TIF) [file pcbi.1000487.s007.tif]

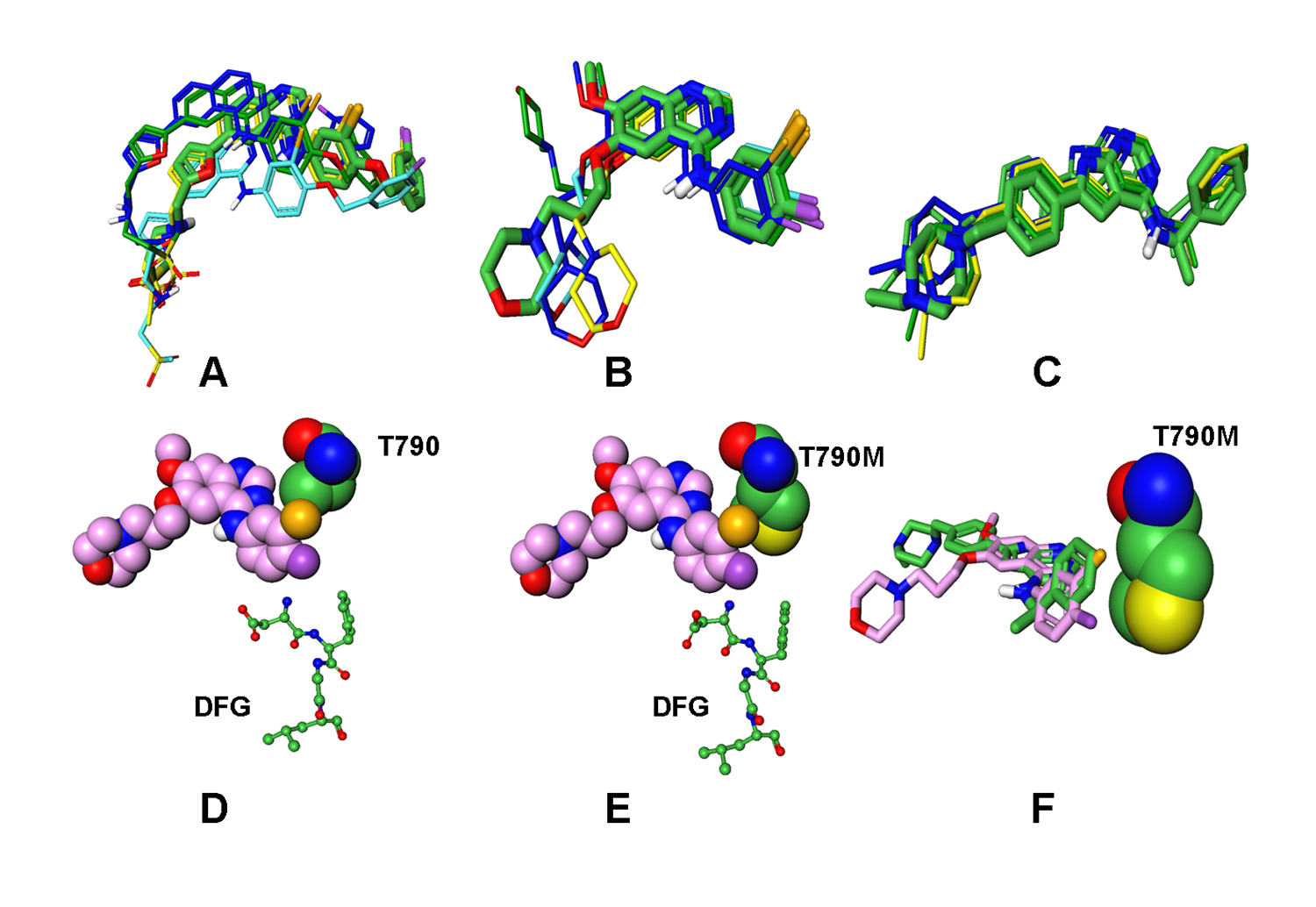

Supplement: Figure S8 — The Predicted Binding Modes of Lapatinib, Geftinib and AE788. The crystallographic conformations of Lapatinib (A), Geftinib (B), and AE788 (C) are shown in default colors (bold, stick). The crystal structure of the inhibitor is superimposed with the predicted binding poses docked into the inactive EGFR-WT (yellow stick), active EGFR-WT (blue stick), EGFR-T790M (light blue stick) and EGFR-L858R (dark green stick). (D) A close-up of interactions between Geftinib and T790 in the WT EGFR crystal structure. (E) A close-up of interactions between Geftinib and T790 in the WT EGFR crystal structure. (F) A close-up of binding modes and interactions formed by Geftinib (in s blue) and AE788 (in default colors) in the hydrophobic pocket of the T790M EGFR mutant. The phenethylamine substituent of AE788 and chlorine-substituted aniline in Geftinib occupy a similar space in the hydrophobic pocket of the T790M EGFR mutant. (1.00 MB TIF) [file pcbi.1000487.s008.tif]
