# Supplementary material for: Hierarchical Modeling of Activation Mechanisms in the ABL and EGFR Kinase Domains: Thermodynamic and Mechanistic Catalysts of Kinase Activation by Cancer Mutations
Source: PLoS Comput Biol. 2009 Aug 28;5(8):e1000487. doi: 10.1371/journal.pcbi.1000487 (PMC2722018; doi:10.1371/journal.pcbi.1000487)
Supplement: Table S1 — A comparison of the experimental and computed binding affinities for the EGFR inhibitors. (0.03 MB DOC) [file pcbi.1000487.s009.doc]

**Table S1. A comparison of the experimental and computed binding affinities for the EGFR inhibitors ***

| **Kinase inhibitor** | **Lapatinib** | **Geftinib** | **AEE788** |
| --- | --- | --- | --- |
| **Crystal Structure (WT)** | 1XKK | 2ITY | 2J6M |
| **Experimental inhibition constant (WT)** | 3.0 | 35.3 | 5.3 |
| **Computed binding free energy (WT)** | -12.5 | -9.2 | -9.8 |
| **Crystal Structure (L858R)** | UND | 2ITZ | 2ITT |
| **Experimental inhibition constant(L858R)** | UNK | 2.4 | 1.1 |
| **Computed binding free energy(L858R)** | -3.8 | -9.5 | -10.0 |
| **Crystal Structure (T790M)** | UND | UND | 2JIU |
| **Experimental inhibition constant (T790M)** | UNK | 4.6 | 27.6 |
| **Computed binding free energy (T790M)** | -3.6 | -9.2 | -8.5 |

* The experimental inhibition dissociation constants are in nM and obtained from [52].

Unknown inhibition dissociations constants are designated as UNK and undetermined crystal structures are designated as UND. The computed binding free energies are in kcal/mol
