# Supplementary material for: Hierarchical Modeling of Activation Mechanisms in the ABL and EGFR Kinase Domains: Thermodynamic and Mechanistic Catalysts of Kinase Activation by Cancer Mutations
Source: PLoS Comput Biol. 2009 Aug 28;5(8):e1000487. doi: 10.1371/journal.pcbi.1000487 (PMC2722018; doi:10.1371/journal.pcbi.1000487)
Supplement: Table S2 — Structure Preparation Details for MD and TMD Simulations of the ABL/EGFR Kinase Domains. (0.03 MB DOC) [file pcbi.1000487.s010.doc]

**Table S2. Structure Preparation Details for MD and TMD Simulations of the ABL/EGFR Kinase Domains**

| **Protein Kinase Structure** | **Total # of atoms** | **Protein atoms** | **Ions** | **Water atoms** |
| --- | --- | --- | --- | --- |
| ABL-WT(1IEP,1M52) | 51403 | 4414 | 45 | 46944 |
| ABL-T315I (1IEP, 2Z60) | 51399 | 4416 | 45 | 46938 |
| ABL-L387M (1IEP,1M52) | 51395 | 4409 | 45 | 46941 |
| EGFRWT (1XKK, 2J6M) | 49169 | 4572 | 41 | 44556 |
| EGFR-T790M(1XKK,2JIU) | 49172 | 4575 | 41 | 44556 |
| EGFRL-858R (1XKK,2ITT) | 49172 | 4577 | 42 | 44553 |
