# Supplementary material for: Hierarchical Modeling of Activation Mechanisms in the ABL and EGFR Kinase Domains: Thermodynamic and Mechanistic Catalysts of Kinase Activation by Cancer Mutations
Source: PLoS Comput Biol. 2009 Aug 28;5(8):e1000487. doi: 10.1371/journal.pcbi.1000487 (PMC2722018; doi:10.1371/journal.pcbi.1000487)
Supplement: Table S3 — Structure Preparation Details for MD Simulations of the ABL and EGFR Complexes. (0.03 MB DOC) [file pcbi.1000487.s011.doc]

**Table S3. Structure Preparation Details for MD Simulations of the ABL and EGFR Complexes.**

| **Protein Kinase Structure** | **Total # of atoms** | **Protein atoms** | **Ions** | **Water atoms** |
| --- | --- | --- | --- | --- |
| ABL-WT(2FO0) | 50049 | 7370 | 40 | 42639 |
| ABL-T315I (2FO0) | 50051 | 7375 | 40 | 42636 |
| ABL-WT(1OPL) | 41622 | 5837 | 34 | 35751 |
| ABL-T315I (1OPL) | 41639 | 5842 | 34 | 35763 |
| EGFR-WT (2GS7) | 57453 | 9347 | 46 | 48060 |
| EGFR-T790M (2GS7) | 57459 | 9353 | 46 | 48060 |
